# Two-Dimensional Theranostic Nanomaterials in Cancer Treatment: State of the Art and Perspectives

**DOI:** 10.3390/cancers12061657

**Published:** 2020-06-22

**Authors:** Iruthayapandi Selestin Raja, Moon Sung Kang, Ki Su Kim, Yu Jin Jung, Dong-Wook Han

**Affiliations:** 1 BIO-IT Foundry Technology Institute, Pusan National University, Busan 46241, Korea; rajaselestin@pusan.ac.kr; 2 Department of Cogno-Mechatronics Engineering, College of Nanoscience & Nanotechnology, Pusan National University, Busan 46241, Korea; mskang7909@gmail.com; 3 Department of Organic Materials Science and Engineering, College of Engineering, Pusan National University, Busan 46241, Korea; 4 Research Centre for Advanced Specialty Chemicals, Division of Specialty and Bio-based Chemicals Technology, Korea Research Institute of Chemical Technology (KRICT), Ulsan 44412, Korea

**Keywords:** two-dimensional nanomaterials, cancer theranostics, tumor, biomedical imaging, in vitro and in vivo biological applications

## Abstract

As the combination of therapies enhances the performance of biocompatible materials in cancer treatment, theranostic therapies are attracting increasing attention rather than individual approaches. In this review, we describe a variety of two-dimensional (2D) theranostic nanomaterials and their efficacy in ablating tumors. Though many literature reports are available to demonstrate the potential application of 2D nanomaterials, we have reviewed here cancer-treating therapies based on such multifunctional nanomaterials abstracting the content from literature works which explain both the in vitro and in vivo level of applications. In addition, we have included a discussion about the future direction of 2D nanomaterials in the field of theranostic cancer treatment.

## 1. Introduction

### 1.1. Two-Dimensional Theranostic Nanomaterials

The term theranotheranostic refers to a comprehensive effort that integrates diagnostics and therapy in a single nanoplatform [1,2]. Nanotheranostic harnesses the capabilities of nanotechnology, enhancing therapeutic efficacy and diagnosing ability with a marked difference compared to other currently available diagnoses and therapies, including chemotherapy [3], immunotherapy [4] and radiotherapy [5]. Though numerous classes of theranostic nanomaterials have been developed for cancer treatment, two-dimensional (2D) nanomaterials and their nanocomposites have been reported to exhibit remarkable advantages in cancer diagnosis and therapy owing to their ultrathin planar nanostructure and intriguing physiochemical properties. The ultrahigh specific surface area rendered by the large lateral size and ultrathin thickness, electron confinement without interlayer interactions, and maximum mechanical flexibility are unique properties of 2D nanomaterials, which differentiate them from their bulk counterparts and other types of nanomaterials such as zero-, one-, and three-dimensional networks [6]. The ultrathin planar nanostructure of these 2D bio-nanosystems provides numerous anchoring sites for therapeutic drug molecules. 

Among these 2D nanomaterials, MXene [7,8], transition metal dichalcogenide (TMDC) [9,10,11,12,13], black phosphorus (BP) [14,15,16,17], graphene oxide (GO) [18,19,20,21], manganese dioxide (MnO_2_) [22,23,24], and palladium (Pd) [25,26,27] have attracted tremendous attention in cancer theranostics [28,29,30]. Generally, 2D inorganic nanoparticles are synthesized either by bottom-up or top-down approaches. The bottom-up methods include organic ligand-assisted growth, 2D template-confined growth, seeded growth, small molecules and ions mediated synthesis, hydro-/solvothermal methods, crystal phase transformation, biological synthesis, and nanoparticle assembly. Mechanical compression, exfoliation, and nanolithography are the methods of top-down approaches [31].

MXenes, which denote the family of 2D transition metal carbides, carbonitrides, and nitrides, have emerged as potential nanocarriers with the intrinsic property of photothermal-conversion in cancer hyperthermia. The temperature point in the surrounding of MXene is elevated upon external irradiation by a near-infrared (NIR) laser with an excitation wavelength at 808 nm. It has been reported that cancerous cells are more sensitive to heat than normal healthy cells. In this context, MXenes have proved capable candidates in the field of biomedicine [28]. Generally, MXenes are produced by extracting A-element from the layered ternary carbides of MAX phases. M indicates an early transition metal, whereas A denotes an A group element in the periodic table. X can be either C or N. There are reports that MXenes exhibit excellent hydrophilicity, metallic conductivity, and mechanical properties [32,33]. It was reported that MXenes, including Ti_3_C_2_Tx and Nb_2_CTx produced by a solvothermal treatment, had five times greater surface areas than the MXenes generated by a HF-etching protocol [34]. In 2016, the first nitride MXene was reported, priduced by the reaction of the MAX phase precursor with molten salts at high temperatures [35]. Limbu et al. described a facile and environmentally benign reduced Ti_3_C_2_Tx MXene prepared through a simple treatment with L-ascorbic acid at room temperature [36]. TMDCs, including MoS_2_, MoSe_2_, WS_2_, WSe_2_, and Bi_2_Se_3_, consist of hexagonal layers of metal atoms sandwiched between two layers of chalcogen atoms. Several research groups have explored TMDCs as drug delivery platforms and NIR-absorbing agents for cancer combination therapy [37]. Many methods, such as direct solvothermal synthesis, chemical vapor deposition, mechanical and chemical exfoliation, and thermal ablation, have been adopted for the scaled-up production of TMDC nanosheets [38]. TMDCs such as NbS_2_, MoS_2_, and WS_2_ were synthesized by the vapor-phase reaction of their respective metal chloride salts at 800–850 °C [39]. Yu et al. have demonstrated the mechanism of deposition in TMDC systems by studying the removal of MoS_2_ nanosheets from the precursor MoCl_5_ in the presence of sulfur gas by the low-pressure chemical vapor deposition reaction [40]. They stated that the rate-limiting step is governed by the relative partial pressure of the MoS_2_ gas and the vapor pressure of the growing MoS_2_ NSs. It was proved that mono-, bi- and tri-layer MoS_2_ nanosheets could be generated if the oxygen plasma treatment is controlled with durations of 90, 120, and 300 s, respectively, prior to its growth [41]. 

Due to the large surface area by volume and intrinsic high NIR absorbance, nanographene oxide (NGO) nanosheets act as the carrier for photosensitizers and photothermal agents as well [42]. They can load many water-insoluble drugs with π–π hydrophobic interactions. They display remarkable theranostic behavior in combinatorial photodynamic and photothermal (PDT/PTT) treatment under 808 nm laser irradiation and hence have been widely used in cancer treatment [43]. It has also been reported that the mass extinction coefficient of GOs is relatively larger than that of gold nanorods. Owing to the fact they are economically inexpensive, GOs have found many more applications in nanomedicine and electrical devices than carbon nanotubes [44]. Li et al. obtained either a monolayer or a few layers of graphene NSs from worm-like graphite in 1-methyl-2-pyrrolidinone suspension using a facial liquid phase exfoliation procedure [45]. Ramesha et al. produced reduced graphene oxide by reducing the exfoliated graphene oxide, which had a high surface area lacking a high negative surface charge [46]. Graphene oxide produced by the Hummers method from flake graphite was reported to interrupt the conjugation in the graphene plane and produce oxygen-containing functional groups, including epoxide, hydroxyl, and carbonyl on its surface [47,48]. Exfoliated 2D MnO_2_ nanosheets with ultrathin thickness were reported to exhibit ultrasensitive responsibility to the tumor microenvironment and release Mn^2+^ in response to mild acidic conditions. Solid tumors are metabolically different from healthy tissues in many ways. They produce an excessive amount of lactic acid and H_2_O_2_ due to the upregulated glycolytic metabolism during tumorigenesis [49,50]. Hence, the metabolism and excretion of the MnO_2_ nanosheets could be facilitated during the theranostic tumor treatment [51]. Omomo et al. reported the synthesis of exfoliated layered manganese oxide (H_0.13_MnO_2_·H_2_O) dissolved in tetrabutylammonium hydroxide solution following a top-down approach [52]. Kazuya Kai et al. showed for the first time a single-step bottom-up approach to synthesize MnO_2_ NSs directly from an aqueous solution of MnCl_2_ [53].

Being a most stable allotrope of phosphorous, black phosphorous (BP) shows a layer-dependent energy band spanning from a bulk value (0.3 eV) to a monolayer value (2.0 eV). It has been reported that BP nanosheets exhibit a high photothermal conversion efficiency with a significant NIR extinction coefficient. Further, BPNSs are biocompatible as the final degradation products of BP, such as phosphonate and phosphate, are non-toxic [54]. BPNSs possess a large surface area with a folded plane configuration and act as an efficient drug delivery candidate [55]. Owing to their unique optical, electronic, and mechanical properties, BPNSs have found several versatile biomedical applications. As BP NSs generate singlet oxygen species in the entire visible light region, they could be employed for photodynamic therapy [56]. Brent et al. synthesized a few-layered BP NSs for the first time via the liquid exfoliation procedure of BP in N-methyl-2-pyrrolidone [57]. Smith et al. produced BP NSs (>3 μm^2^) from red phosphorus directly on a silicon substrate using a chemical vapor deposition method [58]. Because of their strong and well-defined near-infrared (NIR) surface plasma resonance properties and high photothermal stability, palladium (Pd) nanosheets and their nanostructures have been widely used as photo-based theragnostic agents [59]. In 2009, Siril et al. synthesized ultrathin Pd NSs with a thickness of about 2 nm purging carbon monoxide for the first time [60]. The literature reports revealed that the thickness of Pd NSs could be limited to less than 10 atomic layers by the influence of CO [61]. The shape of Pd NSs was extremely stable on exposure to NIR radiation compared to silver and gold nanostructures. Pd NSs, exhibiting a thickness of 2.3 nm and an average edge length of 124 nm, were produced by reducing palladium (II) acetylacetonate in the presence of aqueous ascorbic acid solution [62]. Some other 2D nanomaterials based on boron and gold are available with limited biomedical applications [63,64]. In the present review, we have compiled in vitro and in vivo cancer-treating multifunctional theranostic application of well-explored 2D theranostic nanomaterials. Many reviews have been published, so far, based on 2D theranostic nanomaterials demonstrating various therapies and treatments. They have listed theranostic application of such nanomaterials with respect to biomedical imaging, photothermal conversion efficiency, and dimensional values, including size and thickness of the nanomaterials [65,66,67]. Some literature showed their biological effects randomly either with in vitro or in vivo treatment [65,68]. In the current review, we have presented the theranostic application of 2D nanomaterials collecting only the piece of literature works, which demonstrate both in vitro and in vivo biological effects. 

### 1.2. Theranostic Properties of 2D Nanomaterials in Cancer Treatment

**Figure 1:**
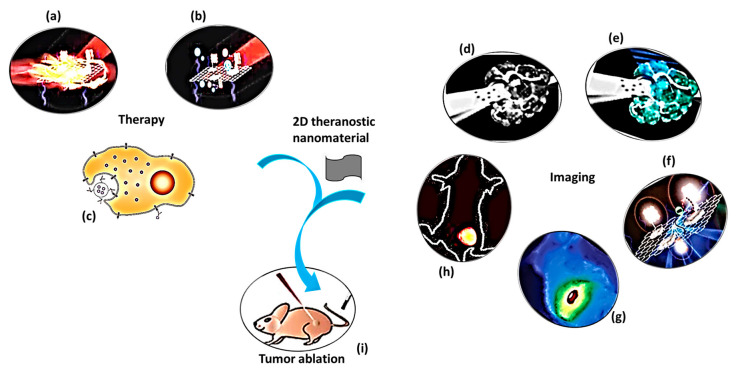
A schematic diagram showing theranostic treatments of 2D theranostic nanomaterials such as photothermal therapy (**a**), photodynamic therapy (**b**) and chemotherapy (**c**) with their astonishing imaging properties, including magnetic resonance (**d**), photoacoustic (**e**), fluorescence (**f**), infra-red thermal (**g**), and upconversion luminescence imaging (**h**) to eradicate cancer tumor (**i**) effectively. The images (**a**), (**b**), (**f**), and (**i**) were adapted with permission from [79]. The image (**c**) was adapted with permission from [80]. The images (**d**) and (**e**) were adapted with permission from [81]. The image (**g**) was adapted with permission from [37]. The image (**h**) was adapted with permission from [82].

The purpose of theranostic therapy is not only enhancing therapeutic effects but also reducing side effects and improving targeting ability. Due to exceptional theranostic photothermal, photodynamic and chemotherapies, 2D theranostic nanomaterials express magnetic resonance [22], photoacoustic [69], fluorescence [70,71], and upconversion luminescent imaging ability [72,73], and sometimes lead to targeted drug delivery [74] (Figure 1). The roles of 2D nanostructured materials in cancer treatment by both in vitro and in vivo studies are listed in Table 1.

Photothermal therapy (PTT) is an emerging treatment method to eradicate cancer tumors, in which light energy is converted into heat energy by using NIR-absorbing agents [75,76]. The use of NIR light triggers a high temporal and spatial control of local heating, minimizing adverse side effects. Two essential features viz. reduced tissue scattering, and high photothermal-conversion efficiency need to be followed to achieve an effective tumor-tissue ablation during NIR irradiation. NIR light can be further classified into two, i.e. NIR-I and NIR-II bio windows with the wavelength range of 750–1000 nm and 1000–1350 nm, respectively [32,77]. During NIR-triggered photothermal treatment, local hyperthermia is produced elevating temperature of the tumor microenvironment more than 42 °C, which is enough to ablate the tumor. Also, PTT bears more significant advantages with minimal invasiveness and a high selectivity than conventional cancer treatment strategies, including simple operation [78]. 

Photodynamic therapy (PDT) is an alternative high-efficient cancer treatment approach, which generates a large amount of singlet oxygen (^1^O_2_) from activatable photosensitizers (PS) and subsequently causes apoptosis or necrosis of cells [83,84,85]. Though PTT and PDT therapies are efficient in cancer treatment, some drawbacks are encountered. PTT, sometimes, causes non-specific cellular damage to healthy tissues because a few portions of NIR laser light travel into some healthy tissues while treating tumor tissues. Likewise, the therapeutic effects of PDT are also limited due to the insufficient oxygen supply and limited penetration depth of visible or ultraviolet light sources. In such circumstances, the impact on tumor growth of a single treatment by either PTT or PDT becomes unsatisfactory. Hence, the researchers introduced the integration of PTT and PDT into one system to improve the therapeutic efficacy of studied nanomaterials [38]. Whenever PSs are administered for the PDT treatment, PSs are retained in tumor tissues at a large quantity because the tumor cells possess an inadequate lymphatic system. In contrast, healthy tissues eliminate them over time. Hence, localized activation by NIR irradiation makes PDT a selective treatment for killing cancer cells. The localized oxidative photodamage triggered by the therapy induces three main mechanisms of cell death at the tumor site. They are apoptosis, necrosis, and autophagy, which are accompanied by the induction of an acute local inflammatory reaction to remove dead cells and restore healthy tissue. PDT is a highly controllable therapy owing to a short-range action of singlet oxygen (^1^O_2_) with a lifetime of ∼40 ns [86]. Apart from NIR-mediated PDT, alternative strategies such as X-ray PDT and sonodynamic PDT were also developed by the researchers to treat cancer. Sonodynamic therapy (SDT) could activate a sonosensitizer through the sonoluminescence process, pyrolytic reaction, or acoustic cavitation effects [87]. The advantageous property of SDT over PDT is the higher tissue penetration depth [88]. In contrast to conventional PDT, X-ray PDT is essentially a combination of RT and PDT, the key factors of which include X-ray dose, the concentration of O_2_, and the efficiency of the intersystem crossing. X-ray PDT is effective in causing oxidative degradation of unsaturated lipids and surface proteins, short-term cell necrosis, and DNA damage [89]. 

Tumor imaging technologies, including magnetic resonance (MR) imaging and photoacoustic (PA) imaging, are related to theranostic therapies, which are helpful for the accurate diagnosis of cancer. MR images are clear with subtle changes due to their excellent resolving power in soft tissues. In contrast, PA imaging produces sharp contrast tissue images as a non-invasive and non-ionizing biomedical imaging technique. Some researchers combine different imaging methods to achieve accurate and early diagnosis of cancer [78]. As photosensitizers are susceptible to photo-bleaching and self-destruction upon prolonged light exposure, the development of novel PS nanocomplexes without fluorescence quenching is necessary for theranostic treatment approaches [79]. Upconversion luminescence (UCL) imaging for tumor cells has attracted substantial attention in recent years owing to the unique properties of upconversion nanomaterials, which minimize the background interference from autofluorescence of biosamples and improve tissue penetration. The upconversion method is an anti-Stokes process whereby two or more low-energy photons from NIR light are absorbed to emit higher energy in the visible region [42]. Sophisticated imaging techniques, including computed tomography, positron emission tomography (PET), and X-rays, are inevitable in cancer diagnosis. When the treatment target is not identified, radiotherapy provides required inputs from imaging for planning the treatment [90]. 

2D theranostic nanomaterials undergo easy surface modification and hence possess a high drug loading capacity for numerous small-molecule anticancer drugs, enzymes, and therapeutic genes. For instance, PEGylated graphene oxide reported by Liu et al. delivered hydrophobic anticancer drug SN38 with therapeutic efficacy than that of FDA-approved SN38 prodrug [91]. The reasons are the specific interactions such as π–π stacking and hydrophobic forces of aromatic ring-containing anticancer drug molecules with the graphene nanosheets. In the acidic environment of cancer tumors, the nanomaterials release the drug molecules due to the protonation effect [92]. Tao et al. conjugated black phosphorus nanosheets with amine-terminated PEG, whereby the loading capacity of DOX was significantly high by 108% w/w. Further, they demonstrated that the interaction between DOX and the black phosphorus could be disrupted by protonation or hyperthermia [15]. The specificity of drug action gains paramount importance in cancer treatment to ensure the minimization of any toxic effects on healthy cells. It was discovered that cancers could be hematologic or solid tumors, and hence different strategies need to be developed for each type of cancer. The distinctive pathophysiological features of tumor tissue are helpful for targeted drug delivery. Tumor-associated antigens (TAAs) such as folate, low-density lipoprotein, and gonadotropin/luteinizing hormone-releasing hormone receptors are the specific proteins, which are highly expressed over the cancer cells [93,94]. When TAAs are conjugated with theranostic 2D nanomaterials, they would apparently enhance tumor therapy, imaging, and drug delivery along with tumor specificity. 

## 2. Application of 2D Nanomaterials

### 2.1. MXene Nanosheets

**Figure 2:**
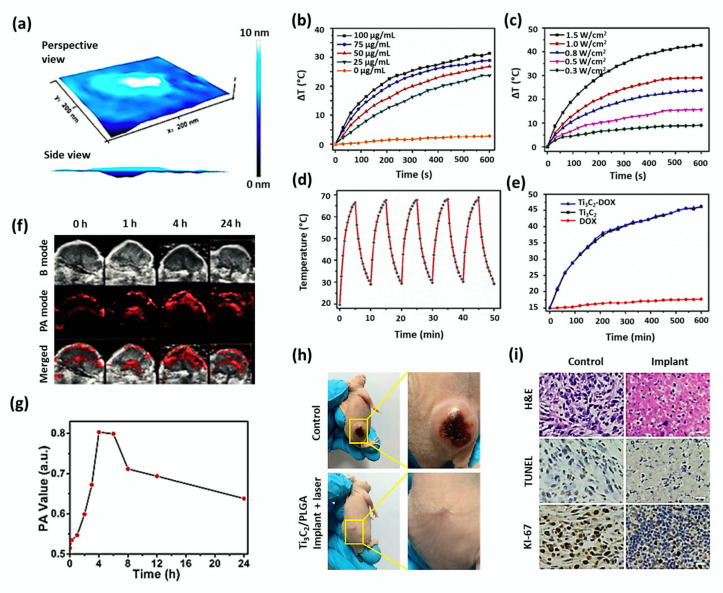
(**a**) 3D AFM image of Ti_3_C_2_ nanosheets. (**b**) Photothermal profile of Ti_3_C_2_ nanosheets in water. Adapted with permission from [81]. (**b**) at various concentrations of 0–100 μg/mL under irradiation (808 nm and 1.0 W/cm^2^) and (**c**) under different laser power densities of 0.3–1.5 W/cm^2^ at concentration of 50 μg/mL. (**d**) Photostability test performed in a solution containing Ti_3_C_2_ nanosheets under irradiation (808 nm, 1.5 W/cm^2^). (**e**) In vitro photothermal performance of Ti_3_C_2_, Ti_3_C_2_-DOX, and free DOX solutions under laser irradiation (808 nm, 1.0 W/cm^2^). Adapted with permission from [95]. (**f**) In vivo 2D B-mode ultrasound (US) imaging, PA imaging, and merged US and PA images of tumor after intravenous administration of Ti_3_C_2_-SP nanosheets at different time duration of 1–24 h. (**g**) The corresponding quantitative changes in PA signal intensity within the tumor have been shown. Adapted with permission from [28]. (**h**) Photographs of 4T1 tumor-bearing mice from the control group and PLGA/ Ti_3_C_2_ implant+NIR laser group on the 16th day of post-injection. (**i**) H&E, TUNEL, and Antigen Ki-67 immunofluorescence staining of tumor tissues of control and PLGA/Ti_3_C_2_ implant+NIR laser group after in vivo photothermal treatment (scale bar: 50 µm). Adapted with permission from [77].

Ultrathin Ti_3_C_2_ nanosheets show strong absorption and conversion efficiency of NIR laser irradiation (808 nm) owing to the localized surface plasmon resonance effect of Ti_3_C_2_ nanosheets of semimetal character [77]. Dai et al. studied the cancer-killing effects of MnO_x_/Ti_3_C_2_ nanosheets with their high photothermal-conversion efficiency of 22.9% [81]. PA signal was enhanced depending on time as the nanocomposite accumulated into the tumor via the typical EPR effect at 24 h of post-injection. The hematoxylin and eosin (H&E) staining, terminal deoxynucleotidyl transferase-mediated dUTP nick end labeling (TUNEL) results demonstrated that MnO_x_/Ti_3_C_2_-SP+NIR group treated mice showed higher necrosis of tumor cells compared to other three groups. A three-dimensional AFM (Atomic Force Microscope) image of ultrathin Ti_3_C_2_ nanosheets has been shown in Figure 2a. Liu G et al. synthesized a multifunctional nanoplatform (Ti_3_C_2_-DOX), the surface of which was modified by layer-by-layer assembly with DOX and hyaluronic acid [95]. As shown in Figure 2b–e, aqueous dispersion of Ti_3_C_2_ elevated the temperature depending on its concentration (0–100 μg/mL) under laser irradiation (808 nm, 0.8 W/cm^2^). A significant increase in temperature was observed for the increasing laser power densities from 0.3 to 1.5 W/cm^2^. Further, it was found that the photothermal effect was not obviously altered after surface modification of nanosheets by DOX and hyaluronic acid. The nanocomposite showed remarkable properties such as tumor-specific accumulation, effective cancer cell killing, and tumor tissue destruction through in vitro and in vivo photothermal, photodynamic, and chemotheranostic therapy.

Ultrathin Ti_3_C_2_ MXene nanosheets were prepared from Ti_3_AlC_2_ powders by a two-step exfoliation procedure [28]. The obtained Ti_3_C_2_ nanosheets had an average planar size of around 120 nm and a thickness of about 0.9 nm. The surface of nanosheets was modified by SP to guarantee a high dispersity and easy transport within the blood vessels and to load DOX mostly for effective cancer treatment. The nanocomposite (DOX@Ti_3_C_2_-SP) was investigated for its theranostic effects of photothermal therapy and chemotherapy subjecting 4T1 breast cancer cell line (in vitro) and 4T1 breast tumor-bearing female nude mice (in vivo). When incubated with 4T1 cells, it was found that DOX@Ti_3_C_2_-SP could be endocytosed into the cells and release the loaded DOX drugs at the intracellular level. When the contrast-enhanced PA images were recorded at given time intervals, it was found that Ti_3_C_2_-SP nanosheets (15 mg/kg) could accumulate in the tumor tissue gradually, providing a significant contrast enhancement in PA imaging (Figure 2f). A shown in Figure 2g, the quantitative determination of PA signal intensity revealed a signal decay due to the gradual excretion of accumulated Ti_3_C_2_-SP nanosheets from the tumor tissues. NIR irradiation in DOX@Ti_3_C_2_-SP treated cells assisted in destroying cancer cells at large quantities when compared to other samples, including Ti_3_C_2_-SP+laser, DOX@Ti_3_C_2_-SP, DOX only, laser only and control. Pathological and immunohistochemical analyses revealed that DOX@Ti_3_C_2_-SP+laser enhanced cell apoptosis and necrosis significantly when compared to other groups due to its useful dual functionality. The DOX@Ti_3_C_2_-SP+laser achieved a complete tumor eradication without re-occurrence, while others showed a partial tumor ablation. 

Lin et al. studied the influence of photothermal conversion effects poly(lactic-co-glycolic acid) modified Ti_3_C_2_ nanosheets (PLGA/Ti_3_C_2_-SP+laser) in 4T1 tumor-bearing Kunming nude mice [77]. At 4 h of post-intravenous injection of nanosheets (20 mg/kg), the laser irradiation increased surface tumor temperature from 30 °C to 58 °C. The tumor-bearing mice treated with PLGA/Ti_3_C_2_ implant+laser were recovered without re-occurrence of the tumor, even after the observation period of 16 days, whereas the control group had a notable increase of the tumor volume (Figure 2h). The successful destruction of tumor cells by PTT using PLGA/ Ti_3_C_2_ implant was demonstrated from H&E and TUNEL staining of tumor sections harvested after the treatment. Further, Ki-67 antibody staining proved a high in vivo antiproliferative activity by the implant (Figure 2i).

The theranostic effects of photothermal conversion and bioimaging of Ti_3_C_2_ MXenes integrated with noble metal (Au) were investigated for cancer treatment [96]. Ti_3_C_2_@Au-PEG nanocomposite was synthesized by seed induced growth method. Due to surface modification with Au, the optical absorption of the nanocomposite in the near-infrared region was enhanced. MTT assay revealed that Ti_3_C_2_@Au-PEG nanocomposites had no potential toxicity effect against 4T1 cells at a higher concentration of 100 μg/mL, but they killed cancerous cells abundantly at the level of 50 μg/mL only when combined with NIR irradiation. PTT followed by X-ray therapy was carried out by injecting Ti_3_C_2_@Au-PEG intravenously into female BALB/c mice bearing 4T1 tumor.

PTT alone with or without the injection of nanocomposite showed no significant inhibition effect, whereas the combination of X-ray/ NIR irradiation showed a better tumor inhibition effect taking advantage of strong optical performance and X-ray attenuation of Au. Histological examination showed that no noticeable abnormality was observed in the major organs of all healthy mice investigated.

A 2D Nb_2_C-based nanocomposite (CTAC@Nb_2_C-MSN-PEG-RGD) was demonstrated showing enhanced chemo-photothermal cancer therapy through NIR-II bio-window (1000–1350 nm), which has a higher tissue penetrating ability than the NIR-I bio-window (750–1000 nm) [97]. The nanocomposite CTAC@Nb_2_C-MSN-PEG-RGD consisted of 2D Nb_2_C MXenes with mesoporous-silica shells on the surface and stabilized by PEG and conjugated with RGD (Arg-Gly-Asp) sequence to bind with integrin α_v_β_3_ receptor, which is overexpressed on cancer cell membranes assisting for targeted drug delivery.

The mesoporous silica in the composite provided saline chemistry and acted as the chemotherapeutic agents along with Nb_2_C MXenes, whereas CTAC micelles encapsulated the whole composite. CTAC@Nb_2_C-MSN-PEG-RGD+laser group exhibited higher cytotoxicity against U87 brain cancer cells as compared to CTAC@Nb_2_C-MSN-PEG-RGD without laser group. It was found that CTAC@Nb_2_C-MSN-PEG-RGD showed efficient in vivo targeting efficacy than CTAC@Nb_2_C-MSN-PEG (without RGD) into U87 subcutaneous tumor-bearing mice. It was reported that there were no changes in body weights of tumor-bearing mice even after different treatments. The tumor-bearing mice in the group of CTAC@Nb_2_C-MSN-PEG-RGD+laser enhanced tumor suppression effect eradicating the tumors. Their tumor inhibition percentage (92.37%) was significantly higher than CTAC@Nb_2_C-MSN-PEG-RGD chemotherapeutic groups without laser (35.96%). 

PVP conjugated niobium carbide nanocomposite (Nb_2_C-PVP) was prepared for effective photothermal therapy to treat cancer [32]. Nb_2_C NSs exhibited a high photothermal conversion efficiency as 36.4% and 45.65% at NIR-I and NIR-II windows, respectively. It was found that all the treated mice survived over 50 days during in vivo photothermal treatment. The tumors of two treated mice groups (Nb_2_C-PVP+NIR-I and Nb_2_C-PVP+NIR-II) were entirely removed by PTT treatment without further re-occurrence. 

### 2.2. TMDC Nanosheets

**Figure 3:**
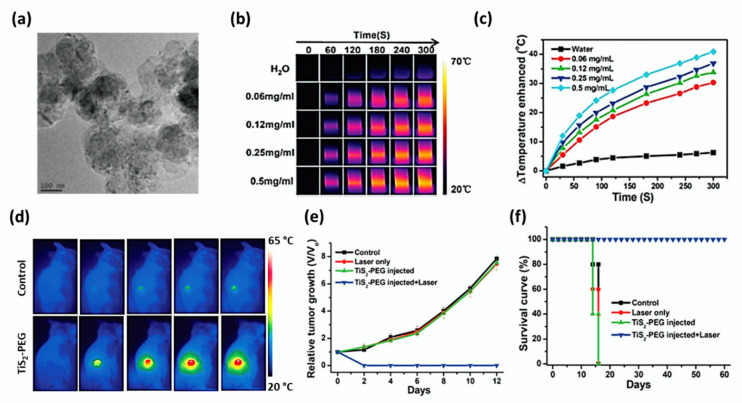
(**a**) TEM image of TiS_2_ nanosheets with scale bar 100 nm. IR thermal images (**b**) and photothermal heating curves (**c**) of pure water and TiS_2_-PEG solutions at different concentrations varying from 0.06 mg/mL to 0.5 mg/mL under 808 nm laser irradiation at a power density of 0.8 W/cm^2^. (**d**) IR thermal images of 4T1 tumor-bearing mice after intravenous injection of 200 μL of TiS_2_-PEG solution (2 mg/mL) at a power density of 0.8 W/cm^2^ for 5 min. (**e**) The relative tumor growth of 4T1 tumors and (**f**) the survival curves of different mice groups (control (saline), laser only, TiS_2_-PEG with or without laser) were indicated with standard deviation (*n* = 5). Adapted with permission from [37].

Qian et al. synthesized TiS_2_ nanosheets following a bottom-up solution-phase approach and subsequently modified using PEG [37]. A TEM image of the TiS_2_ nanosheets is shown in Figure 3a. He proved that TiS_2_-PEG not only served as a photothermal agent, but also acted as a photoacoustic contrast agent, owing to its high NIR absorbance (Figure 3b,c). Due to surface modification, the obtained nanocomposite (TiS_2_-PEG) exhibited excellent stability in various solutions such as saline, serum, and cell medium. When compared to other traditional optical imaging methods, PA imaging is known as a non-invasive imaging modality presenting an increased in vivo imaging depth and spatial resolution. When photoacoustic imaging was conducted at different time points of 0, 2, 4, 8, 12, and 24 h after intravenous injection of TiS_2_-PEG to tumor-bearing mice, strong photoacoustic signals were observed to show the dispersion of blood vessels within the whole tumor. At the concentration of 100 μg/mL of nanosheets, no significant cytotoxicity was noticed to the murine breast cancer cells (4T1) in the absence of irradiation. More 4T1 cells were destroyed under NIR irradiation (808 nm, 0.8 W/cm^2^), suggesting that TiS_2_ nanosheets could serve as active photothermal agents. 

After the intravenous injection of 20 mg/kg of TiS_2_-PEG nanosheets, the 4T1 tumor-bearing mice were exposed to an 808 nm laser at 0.8 W/cm^2^ for 5 min after 24 h. It was found that TiS_2_-PEG+laser irradiation ablated tumors after 1 day of treatment. The black scars left at the primary tumor sites fell off in about 10 days. IR thermal images of cancer after surgery, relative tumor growth, and the survival curves of different groups of mice have been shown in Figure 3d–f. Histological analyses evidenced that TiS_2_-PEG exerted no apparent toxicity to mice at all the concentrations studied. The mice of the TiS_2_-PEG treated group survived over 60 days after PTT, whereas the mice of the control group died within 16 days.

Efficient MoS_2_-CS nanosheets were synthesized as a promising contrast agent in X-ray computed tomography imaging with an apparent X-ray absorption ability of molybdenum (Mo) [98]. The photothermal conversion efficiency of MoS_2_-CS was reported to be 24.37%. The NIR-controllable drug release and the cellular uptake of DOX upon 808 nm NIR irradiation was demonstrated in KB and Panc-1 cancer cells. After incubation of KB cells with MoS_2_-CS-DOX for 2 h, DOX fluorescence signals were observed inside the cells, which indicated the efficient uptake of MoS_2_-CS-DOX by the cells. After irradiation, the red fluorescence signals increased, suggesting that a large number of free DOX molecules were delivered from the intracellular MoS_2_-CS-DOX. Most of the tumor tissues treated with MoS_2_-CS-DOX perish from necrosis, including eosinophilic cytoplasm, karyorrhectic debris, and nuclear damage compared to the control group. For the Panc-1 cells, MoS_2_-CS-DOX+NIR presented a remarkable cell-killing ability at each studied concentration (0–100 μg/mL), due to theranostic hyperthermia and chemotherapy. 

Pan et al. synthesized gadolinium (Gd^3+^)-doped MoSe_2_ nanosheets using a simple liquid-phase method and obtained MoSe_2_(Gd^3+^)-PEG after surface modification by PEG [78]. The obtained nanocomposite had high stability in water, PBS, cell culture medium, and fetal bovine serum. It was reported that Gd^3+^ could be used for producing a strong magnetic resonance imaging effect. The intravenous injection of nanocomposite into the Hep G2 tumor-bearing BALB/c nude mice eliminated the tumor under irradiation at 808 nm for 5 min. Bai et al. produced bovine serum albumin and methylene blue conjugated bismuth telluride nanosheets (BSA-Bi_2_Te_3_/MB) with photodynamic and photothermal properties to treat cancer [38]. BSA was used as an exfoliating agent for the synthesis of Bi_2_Te_3_, which improved the dispersion of nanocomposite in solution. The photothermal conversion efficiency of BSA-Bi_2_Te_3_ NSs was about 45.3%, and the loading content of MB with the stabilized nanosheets was 101.7 μg/mg. Mice bearing U14 tumors were divided into five groups, and the mice group treated with BSA-Bi_2_Te_3_/MB+PDT/PTT was reported to have a moderate growth inhibition effect than the nanocomposite combined with PDT or PTT alone.

### 2.3. Graphene Oxide Nanosheets

Epidermal growth factor receptor (EGFR) is a receptor tyrosine kinase that overexpresses in solid tumors in many organs such as breast, ovarian, bladder, glioma, lung, pancreatic, kidney, and prostate, which makes it an attractive target in cancer treatment. Yang et al. developed a new EGFR targeted drug delivery system, labeled as PEG-NGO-C225/EPI, for the purpose of blocking EGFR growth signal with targeted chemotherapy, and NIR light-mediated phototherapy [100]. The formulated nanocomposite system contained NGO loaded with the anticancer drug (epirubicin, EPI), and anti-EGFR monoclonal antibodies (cetuximab, C255). The in vitro results exposed that the PEG-NGO-C225/EPI drug system could release the active drug to the cytoplasm of target cells depending on the pH-condition. Moreover, it was demonstrated that the conjugation of C225 with PEG-NGO significantly enhanced its ability to downregulate EGFR inducing apoptosis. The treatment of U87 cells with PEG-NGO-C225 instigated a dramatic decrease in EGFR expression, whereas C225 alone only caused a slight decrease. The concentration required for 50% inhibition of cellular growth for free EPI was 15.1 μg/mL, which was slightly larger than that of PEG-NGO-/EPI (13.2 μg/mL). A 2 min of laser irradiation in PEG-NGO-C225/EPI injected mice caused the tumor temperature to rise remarkably up to 88 °C (ΔT = 51 °C), while mice treated with laser alone had increased tumor temperature with ΔT = 7 °C. From fluorescence microscope observation, it was confirmed that a large number of PEG-NGO-C225/EPI deposited at the tumor site due to the multivalent interaction of C225. Further analyses presumed that tumor cell proteins and DNA were severely damaged during the treatment. The black scars in the tumor site fell off at 7 days of post-treatment. 

**Figure 4:**
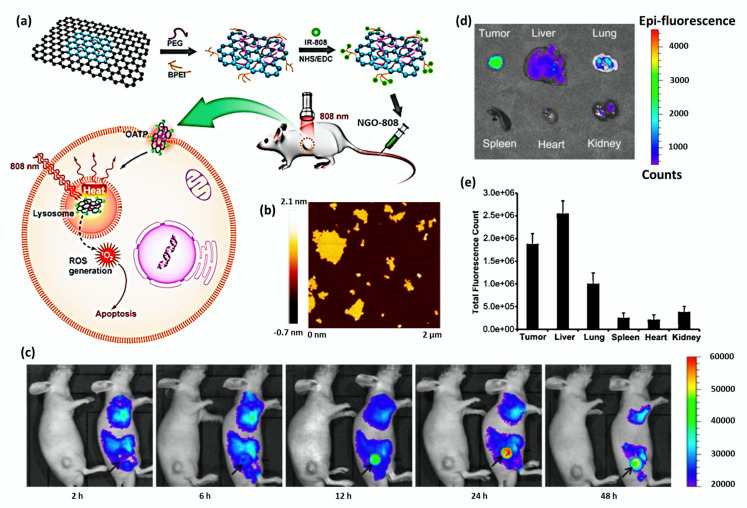
(**a**) A schematic diagram explaining the formation of tumor-targeted NGO-808, which was obtained by grafting NIR photodynamic photosensitizer (IR-808) onto the PEG- and BPEI dual-functionalized nanographene oxide (NGO). PDT/PTT combination therapy was achieved by the enhanced tumor accumulation through IR-808-mediated organic anion transporting polypeptide (OATP) transport under laser irradiation (808 nm). (**b**) AFM image of bare NGO nanosheets. Adapted with permission from [43]. (**c**) In vivo NIR fluorescence images of HeLa tumor-bearing nude mice after intravenous injection of saline (left mouse) or Cy 5.5 labeled Pluronic functionalized NGO (right mouse). The position of the tumor was indicated by a black arrow. (**d**) Ex vivo NIR fluorescence images of major organs and tumors of sacrificed mice showed a high fluorescence signal in tumor tissue, indicating a substantial accumulation of Pluronic coated NGO in the tumor. (**e**) Biodistribution profile of Pluronic functionalized NGO in major organs of mice at 48 h of post-injection (*n* = 3). Adapted with permission from [99].

Luo et al. conjugated a PDT photosensitizer (IR-808) with dual functionalized nanographene oxide (PEG- BPEI- NGO) to produce NGO-808 for studying theranostic effects of PDT/PTT [43]. The schematic diagram of the same and AFM image of NGO has been shown in Figure 4a,b, respectively. Sahu et al. developed methylene blue-loaded Pluronic stabilized nanographene oxide nanocomposite (Pluronic F127-NGO-MB) for cancer therapy. Methylene blue is a hydrophilic and water-soluble phenothiazine derivative, which acts as a promising photosensitizer for photodynamic therapy against microbial cells, viruses, and cancer cells [99]. There were reports that MB has a high quantum yield of ^1^O_2_ generation exhibiting an excitation wavelength range of 600–900 nm possessing low dark toxicity. As NGO was negatively charged with the presence of COOH groups, the positively charged MB was largely loaded onto the nano-Pluronic-coated system through electrostatic interaction. An 80% MB drug was released from the Pluronic F127-NGO-MB drug system in 72 h at pH 5.0, while only 25% of drug release was observed in a similar duration at pH 7.4 during in vitro drug release, indicating the pH-dependent release. As the intracellular lysosomes, endosomes, and extracellular tissues of tumors are acidic in nature, pH-dependent drug release is an advantageous property in cancer therapy. 

NIR fluorescence signals were recorded by injecting Cy 5.5 labeled NGO into HeLa tumor-bearing nude mice. It was found that fluorescence was broadly distributed within the whole body at 2 h and 6 h of post-injection. A strong fluorescence signal was observed at the tumor site after 12 h of post-injection, which indicated the prolonged blood circulation and efficient tumor accumulation of Pluronic coated NGO (Figure 4c). 

Fluorescence intensity of major organs and tumor excised from mice was evaluated using the IVIS 100 imaging system at 48 h of post-injection. As shown in Figure 4d and Figure 4e, the excised tumor tissue showed significantly stronger fluorescence intensity when compared to liver and lung tissues. Meanwhile, the intensity was found very low in the spleen, heart, and kidney. 

Shi et al. demonstrated that functionalized GO-IONP-Au-PEG nanocomposite could serve as an effective PTT agent with molecular and magnetic targeting features [101]. Due to the strong X-ray absorbance of the heavy element (Au), the nanocomposite functioned as an X-ray absorber in CT imaging. The concentration-dependent darkening effect was revealed by T_2_-weighted MR images of the composite acquired by a 3-T MR scanner. Yan et al. designed a photosensitizer-loaded PEGylated graphene oxide (GO-PEG-DVDMS) to treat cancer cells effectively with the enhanced fluorescence/photoacoustic dual-modal imaging and theranostic PDT/PTT therapy [79]. When assessing ROS generation, both free photosensitizer (DVDMS) and GO-PEG-DVDMS exhibited a sharp increase in singlet oxygen sensor green (SOSG) fluorescence intensity in a time-dependent manner suggesting that the DVDMS can generate ROS after being loaded by GO-PEG. In another work, 2-(1-hexyloxyethyl)-2-devinyl pyropheophorbide-α (HPPH) loaded PEG-functionalized graphene oxide (GO-PEG-HPPH) was developed with PDT efficiency to destroy cancer cells [102]. The in vivo distribution and release of the nanocomposite were tracked by fluorescence cell imaging and positron emission tomography (PET) after the radiolabeling of HPPH with ^64^Cu. It was found that the average tumor fractional oxygen saturation (sO_2_) was significantly decreased, and organ damage did not occur in both free HPPH and GO-PEG-HPPH treated mice groups. Gulzar et al. studied the UCL imaging-guided PDT/PTT therapy using a graphene oxide-based nanocomposite (NGO-UCNP-Ce6) to treat cancer cells effectively [42]. The cellular uptake of the sample was investigated using laser scanning upconversion luminescence microscopy. The in vitro and in vivo biological results exposed that the nanocomposite could be used as an encouraging contender for cellular labeling, UCL imaging, and PDT/PTT therapy.

### 2.4. MnO_2_ Nanosheets

**Figure 5:**
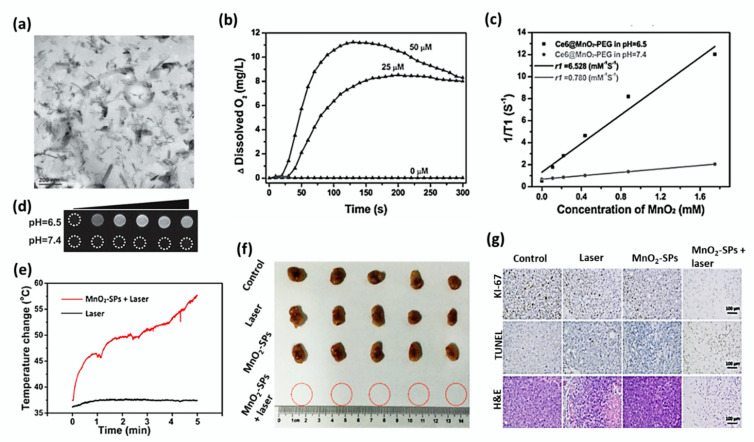
(**a**) Bright-field TEM image of MnO_2_ nanosheets with scale bar 200 nm. Adapted with permission from [111]. (**b**) O_2_ generation efficacy of MnO_2_ nanosheets in H_2_O_2_ solution at different concentrations of Ce6@MnO_2_-PEG (0, 25, and 50 µM). (**c**) The concentration-dependent T_1_ relaxation rates and the longitudinal relaxivities (r1) of the composite measured at different pH 7.4 and 6.5. (**d**) T_1_-weighted MR images of various concentrated Ce6@MnO_2_-PEG nanocomposite at different pH values 7.4 and 6.5. Adapted with permission from [103]. (**e**) Increase in temperature observed at the tumor site of BALB/c nude mice bearing 4T1 tumor xenograft during NIR-triggered PTT treatment after intravenous injection of MnO_2_-SPs nanosheets. (**f**) Digital photos of tumors excised from 4T1 tumor-bearing BALB/c nude mice after 16 days of intravenous injection followed by different treatments. (**g**) Tumor tissues of each group after PTT were stained by Antigen Ki-67 immunofluorescence (upper), TUNEL (middle), and H&E (bottom) (Scale bar: 100 µm). Adapted with permission from [51].

Chen et al. showed the T_1_-weighted MR imaging and pH-sensitive drug release properties of MnO_2_ nanosheets. The bright-field TEM image of MnO_2_ nanosheets has been shown in Figure 5a [111]. The large surface area of exfoliated MnO_2_ NSs provided numerous anchoring points for DOX drug molecules, which were loaded onto the surface of nanosheets via electrostatic interaction and Mn-N coordination bonds. Meanwhile, the loaded cargos could be released very fast in an acidic microenvironment like a cancer tumor, based on the break-up of MnO_2_ [112]. Owing to these properties, MnO_2_ nanosheets assisted in the high accumulation of DOX within the cancer cells and time-dependent pH-responsive intracellular drug release. The acidity-induced break-up of MnO_2_ nanosheets and the DOX-release process were monitored in situ by using MRI. The in vivo tumor imaging and ultrasensitive pH-responsiveness were evaluated using 4T1 tumor-bearing female nude mice. It was found that the acidic environment of tumors substantially promoted the break-up of the carrier and subsequently led to the fast release of the loaded DOX-loaded MnO_2_ nanosheets into the tumor, reflecting the in vitro results. Manganese-based oxides have been demonstrated as contrast agents for T1-weighted MRI with the improved biocompatibilities [113,114,115]. As the Mn atoms in MnO_2_ nanosheets are coordinated with six oxygen atoms in an octahedral geometry, they do not contribute to the longitudinal or transverse relaxation of the protons in water molecules. Meanwhile, disintegration and degradation of MnO_2_ nanosheets release Mn^2+^ giving rise to the enhanced T_1_-MRI performance due to the five unpaired 3d electrons of Mn^2+^ [116,117]. Moreover, the redox reaction between MnO_2_ and GSH occurred in the acidic microenvironment of the tumor has been reported to favor its application in cancer treatment [118].

PEGylated Ce6 loaded MnO_2_ nanosheets (Ce6@MnO_2_-PEG) were proved to be effective in killing cancer cells by PDT, even in the hypoxic microenvironment [103]. The highly reactive MnO_2_ converted endogenous H_2_O_2_ produced by cancer cells into O_2_, as shown in Figure 5b. When MR imaging of Ce6@MnO_2_-PEG solution was conducted at different pH values 6.5 and 7.4, the concentration-dependent brightening effect was observed. The T_1_-weighted MR signal of Ce6@MnO_2_-PEG samples at pH 6.5 was stronger than that of the sample at pH 7.4. The relaxivity (r1) of Ce6@MnO_2_-PEG was found very low as 0.780 mM^−1^ s^−1^ at pH 7.4, whereas the r1 value measured at pH 6.5 increased up to 6.528 mM^−1^ s^−1^ showing a significant difference (Figure 5c,d). The intravenous injection of Ce6@MnO_2_-PEG accumulated largely into the 4T1 tumor of BALB/c mice, and it showed T_1_-weighted magnetic resonance imaging to a great extent as a result. PDT cancer treatment was found effective under a largely reduced dose, owing to the enhanced uptake of nanocomposite and reversed tumor hypoxia.

The paramagnetic Mn component in the nanosheets functions as the contrast agents for MR imaging. Liu et al. characterized soybean phospholipid stabilized MnO_2_ nanosheets (MnO_2_-SPs) for their efficient T_1_-weighted MRI imaging, pH-responsive drug release, and NIR-triggered PTT [51]. The photothermal-conversion studies claimed that MnO_2_ solution (150 μg/mL) rapidly increased the temperature over 50 °C within 5 min while the temperature of deionized water increased by 1 °C only. MnO_2_-SPs exhibited low cytotoxicity against 4T1 cells, even at the concentration of 600 μg/mL. 

However, the nanosheets induced concentration-dependent cell death under 808 nm laser irradiation at the power density of 1.5 W/cm^2^. BALB/c nude mice bearing 4T1 breast tumor were randomly divided into four different groups, including control, laser only, MnO_2_-SPs and MnO_2_-SPs+laser. IR thermal images showed that the tumor surface temperature of the MnO_2_-SPs+laser group significantly increased from 37 °C to 57 °C within 5 min of irradiation. In comparison, the heat of the laser-only group increased only by 1 °C (Figure 5e). 

It was noted that the tumor of the MnO_2_-SPs+laser group was eliminated without the obvious re-occurrence at the end of the evaluation (Figure 5f). Histological analyses including H&E, TUNEL, and antigen Ki-67 immunofluorescence evaluation of tumor tissues showed that the tumors of the other three mice groups except MnO_2_-SPs+laser displayed normal cell morphology and well-organized nuclear structure (Figure 5g).

Fan et al. also studied a similar kind of work subjecting MnO_2_ nanosheets anchored with upconversion nanoprobes (UCSMs) in hypoxic murine breast cancer hc-4T1 tumor cells [50]. The elevated concentrations of endogenous acidic H_2_O_2_ reacted with MnO_2_ of UCSMs to generate O_2_ in situ and thereby enhanced the synergetic PDT/RT efficacy upon NIR/X-ray irradiation. It was reported that the injection of UCSMs not only increased the signal intensity of oxygenated/deoxygenated hemoglobin but also enhanced tumor sO_2_ by about 7%. UCSMs influenced to down-express hypoxia-inducible factor 1-alpha and vascular endothelial growth factor in cells indicating a decreased hypoxia or increased oxygenation. According to in vivo animal studies, though the treatments of UCSMs+NIR and UCSMs+RT inhibited tumor growth, UCSMs+RT+NIR significantly promoted synergetic PDT/RT effects on 4T1 solid tumors and remarkably regressed the substantial tumor volume.

### 2.5. Black Phosphorous Nanosheets

**Figure 6:**
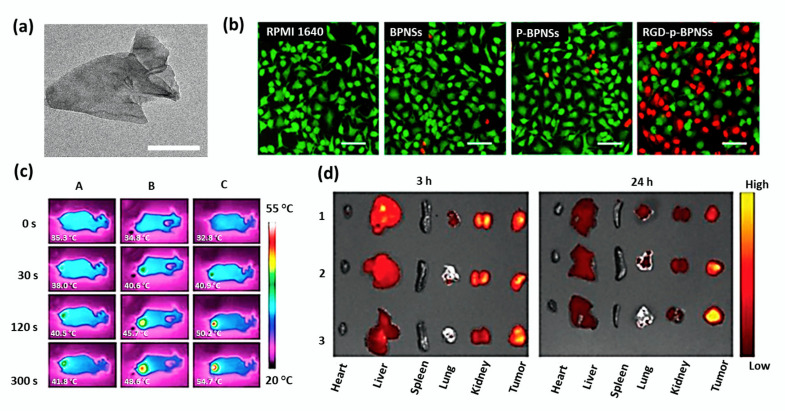
(**a**) TEM image of bare BPNSs with scale bar 50 nm. (**b**) Inverted fluorescence microscope images of A549 cells (scale bar 100 μm) treated with RPMI 1640 cell culture medium, bare BPNSs, p-BPNSs (1-pyrenebutyric acid-modified BPNSs), and RGD-p-BPNSs after 30 min of incubation followed by NIR irradiation laser (808 nm, 1.0 W/cm^2^, 10 min). Propidium iodide (red, dead cells) and Calcein-AM (green, live cells) were used for staining the cells. Adapted with permission from [104]. (**c**) In vivo IR thermal images of mice treated with A: PBS; B: BP@PDA-PEG; C: BP@PDA-PEG-Apt. (**d**) Ex vivo fluorescence images of tumors and major organs captured at 3 and 24 h of post-administration of free DOX (1), BP-R-D@PDA-PEG (2), and BP-R-D@PDA-PEG-Apt (3). Adapted with permission from [54].

For a decade, black phosphorus-based 2D nanomaterials have emerged as excellent inorganic materials in biomedicine due to their biocompatibility, biodegradability, and biosafety. It was reported that the metabolism of BP would not cause certain immune responses [55]. Li et al. demonstrated that RGD-modified p-BPNSs (RGD-p-BPNSs) exhibited targeted photothermal efficacy against A549 cells [104]. The results were analyzed comparing with RPMI 1640 cell culture medium, bare BPNSs, and p-BPNSs (1-pyrenebutyric acid-modified BPNSs). The increased number of dead cells was observed for RGD-p-BPNSs treatment after 30 min of incubation, followed by 10 min of NIR laser. TEM image of bare BPNSs and in vitro photothermal efficiency of RGD-p-BPNSs mediated by NIR laser have been shown in Figure 6a,b.

Permeability glycoprotein (P-gp) siRNA and DOX were employed to develop a multifunctional BP based co-delivery system (BP-R-D@PDA-PEG-Apt) for achieving a targeted gene/chemo/photothermal therapy against multidrug-resistant cancer [54]. It is important to note that P-gp siRNA is responsible for the P-gp mediated multidrug resistance. Polydopamine (PDA) was used in nanocomposite to improve stability and pH-sensitive delivery. The single-stranded oligonucleotide, aptamers (Apts) have emerged as active tumor targeting agents with non-immunogenic tissue penetrating property. The photothermal efficiency of BP@PDA was slightly higher (ΔT = 27.1 °C) than bare BP (ΔT = 24.1 °C), due to PDA coating. Owing to the knockdown of P-gp on the cell membranes, DOX-siRNA-BP NSs led to higher cytotoxicity in MCF-7/ADR cells than DOX alone. The tumor temperature of the BP@PDA-PEG-Apt treated mice group rapidly increased to 54.7 °C under irradiation for 5 min, and ablated tumor effectively. Moreover, the DOX fluorescence was much stronger at the tumor site of the BP-R-D@PDAPEG-Apt treated group indicating an excellent tumor targeting ability. The IR thermal images and ex vivo fluorescence images of tumors and major organs of mice were shown in Figure 6c,d.

Wang et al. synthesized a BP-based small interfering RNA (siRNA) delivery system (BP-PEI-siRNA) to hamper the production of target protein along with photothermal property [56]. It was discovered that PEI could cause the swelling and rupture of endosomes and release the trapped materials into the cytoplasm due to its ‘proton sponge’ effect, and siRNA was reported to regulate the translation of messenger RNA. During irradiation (808 nm for 10 min), the temperature of the BP-PEI solution increased by 17.8 °C, which was significantly higher than that of water solution (ΔT = 3.1 °C) under the same condition. The photothermal property of the BP-PEI-siRNA composite was studied in MCF-7 tumor-bearing nude mice. The mice were divided into four groups viz. PBS (control), BP-PEI+laser, BP-PEI-siRNA only, and BP-PEI-siRNA+laser. The experimental results proved that the combination of photothermal and gene therapy exhibited an excellent tumor ablation effect in vivo. There was no significant organ failures and inflammatory lesion in 20 days, which indicated a long-term safety of the BP-PEI-siRNA mediated treatment.

An organelle-targeting BP nanosheet-based nanocomposite (BP@PDA-Ce6&TPP) was synthesized to treat cancerous cells with PTT/PDT therapy theranostic effects [55]. The BP nanosheets were coated by PDA and subsequently crosslinked covalently by both chlorin e6 (Ce6) and triphenylphosphonium (TPP) through carbodiimide reaction to prepare the nanocomposite. Ce6, a commercial photosensitizer, was used to generate ROS effectively, while TPP was used to target mitochondria via membrane potential. It was found that BP@PDA-Ce6&TPP localized into mitochondria selectively rather than cytoplasm of Hela cells. After laser illumination (660 nm, 0.5 W/cm^2^, 5 min), 50% of the BP@PDA-Ce6&TPP treated cells were killed, which was estimated to be much higher than those treated with BP@PDA-Ce6 (32%) and BP@PDA (3%). When the concentration of BP@PDA in the nanocomposite increased, its therapeutic efficacy was also found increased. A 100 μL of saline solution containing 1 × 10^6^ Hela cells was subcutaneously injected into the mice to generate a tumor. The tumor temperature was increased from 37.6 to 48.1 °C after 10 min of laser irradiation, which was identified when IR thermal imaging was performed at 12 h post-injection of BP@PDA-Ce6&TPP. Organ damage, bodyweight reduction, and inflammatory lesion were not observed in all major organs after the combined PDT/PTT therapy.

BP nanosheet was used as a template to incorporate doxorubicin (D), poly-L-lysine (L), and hyaluronic acid (H) through the layer-by-layer process by the research group of Poudel et al. [105]. Hyaluronic acid acted as a targeting agent for CD44-overexpressing malignant and chemo-resistant tumors cells. The pH-responsive, CD44 receptor-targeted and NIR-triggered photothermal drug release of the nanocomposite were investigated through in vitro MCF-7 and MDA-MB-231 breast cancer cell studies and in vivo MDA-MB-231 tumor-bearing BALB/c mice treatment. The cumulative drug release from BP-DLH was 59% and 77% at pH values 7.4 and 5.5, respectively, which suggested that the nanocomposite followed a low pH-responsive drug delivery. MTT assay revealed that BP-DLH was biocompatible with >80% cell viability in the absence of NIR irradiation. When irradiated by laser (808 nm, 0.8 W/cm^2^, 2.5 min), the nanocomposite acted as a photo transducer and reduced cell viability in a dose-dependent manner. Nearly >95% of cytotoxicity was recorded at 50 μg/mL of the nanocomposite. The tumor section harvested from BP-DLH+NIR mice showed drastic microstructural alterations, including apoptotic condensation and cellular fragmentation, whereas untreated control samples retained their morphology with no observation of nuclear atypia. 

### 2.6. Pd Nanosheets

**Figure 7:**
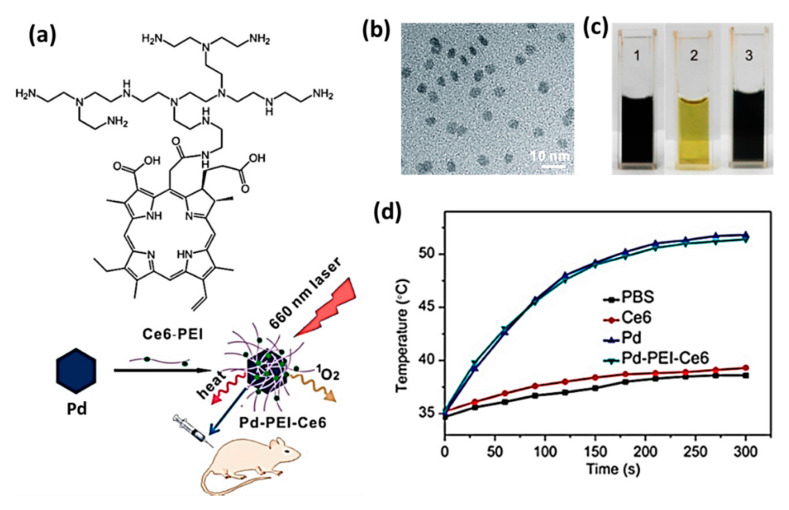
(**a**) A schematic diagram explaining the preparation and photothermal application of pd-PEI-Ce6 (**b**) TEM image of Pd-PEI-Ce6 (**c**) Photograph of solutions containing Pd nanosheets (1), Ce6-PEI (2), and Pd-PEI-Ce6 (3) in PBS at pH 7.4. (**d**) An increase in temperature was observed on the tumor sites of mice treated with PBS, Ce6, Pd, and Pd-PEI-Ce6 after irradiation by 660 nm laser (0.5 W/cm^2^). Adapted with permission from [106].

The Pd nanosheets exhibit a strong UV-vis absorption in the range of 450–800 nm showing a maximum at 660 nm. Zhao et al. prepared a Pd-PEI-Ce6 nanocomposite with theranostic effects of PTT/PDT, which involves in photothermal conversion generating a large amount of singlet oxygen upon irradiation by NIR laser at 660 nm [106]. (Figure 7a). TEM image of Pd-PEI-Ce6 and digital photograph image of different PBS solutions containing Pd nanosheets, Ce6-PEI, and Pd-PEI-Ce6 have been shown in Figure 7b,c. Upon irradiation (0.5 W/cm^2^, 12 min), the increase in temperature was observed up to 44 °C and 42 °C for Pd alone and Pd-PEI-Ce6, respectively, while free Ce6 solution and water had negligible changes (Figure 7d). The in vitro staining experiments to assess the viability of HeLa cells revealed that Pd-PEI-Ce6 treated cells killed more cells, which was observed from more intense red fluorescence than that of Ce6 or Pd nanosheets treated ones.

Tang et al. synthesized ultrasmall sized Pd nanosheets (SPNS) with an average diameter of ~4.4 nm to exhibit superior photothermal efficiency and optimal clearance characteristics [107]. DOX molecules were also loaded on SPNS mainly through Pd-N coordination bonding to achieve effective chemotherapy. The resulting hybrid (SPNS-DOX) was surface-functionalized with reduced glutathione (GSH) to produce the nanocomposite (SPNS-DOX-GSH) with theranostic properties in cancer therapy. The coordinative bonding of DOX on SPNS augmented its accumulation in tumor tissue and therefore reduced the laser power required to achieve effective tumor ablation. SPNS were also reported to serve as pH-responsive drug carriers for efficient DOX delivery. Shi et al. treated malignant tumors using chlorin e6 linked mesoporous silica-coated Pd@Ag nanoparticles (Pd@Ag@mSiO_2_-Ce6) applying for NIR irradiation at different wavelengths 660 nm, 808 nm, or both [108]. Chen et al. studied the photoacoustic effect of four different-sized Pd nanosheets in a concentration-dependent manner and demonstrated the photothermal effect at different NIR irradiation [59]. They proved that smaller-sized Pd nanosheets showed better biocompatibility than the larger-sized Pd nanosheets. It was reported that 80-nm Pd nanosheets altered more biological processes and cellular components in tumor-bearing mice than 5-nm Pd nanosheets. Further, lipid accumulation in liver and inflammatory responses was produced in both the 5- and 80-nm Pd nanosheet treated mice groups.

### 2.7. Other 2D Theranostic Nanomaterials

Liu et al. systematically evaluated PTT/PA therapeutic properties of two-dimensional Au nanorings [109]. Among different sizes of nanorings (25, 50, and 130 nm), 50 nm Au nanoring displayed the highest tumor delivery efficiency. The photothermal conversion efficiency of Au nanorings with thicknesses of 4, 9, and 20 nm was calculated to be 43.5 ± 3.2, 37.9 ± 2.2, and 31.9 ± 2.3%, respectively. The estimated photothermal conversion efficiency was found to decrease with increasing ring thickness under NIR laser irradiation (808 nm, 0.5 W/cm^2^). When 50 nm thiol-terminated poly(ethylene glycol) methyl ether stabilized Au nanorings (HS-PEG@Au) were intravenously injected into U87MG tumor-bearing nude mice, there was a signal enhancement about 7.7 times compared to background signal at 24 h of post-injection.

A new photonic drug delivery platform based on the PEGylated boron nanosheets (B-PEG NSs) was developed by the research group of Ji et al., which released the drug DOX by NIR light and acidic pH [110]. The synthesized nanocomposite had 42.5% of photothermal conversion efficiency and exhibited multimodal imaging properties, including photoacoustic, photothermal, and fluorescence imaging. Under the laser irradiation, B-PEG NSs killed more than 85% of MCF-7 and up to 90% of PC3 cells at a concentration of 200 μg/mL. Blood biochemistry and hematological analyses revealed that B-PEG NSs did not cause apparent infection and inflammation in the treated mice.

## 3. Biosafety of 2D Theranostic Nanomaterials

Generally, inorganic nanomaterials are relatively challenging to degrade and excrete in biological applications. They have a long residence time in living organisms causing adverse effects, including inflammation and tissue cysts [112]. Hence, consideration of the biosafety profile of theranostic 2D nanomaterials is of great importance for their cancer treatment. The toxicity of nanomaterials depends on the complex interaction of several physicochemical properties, such as size, shape, surface modification, oxidative state, dispersion state, synthesis procedure, exposure time, dose, and route of administration [119,120,121].

While evaluating the biosafety of Ti_3_C_2_ NSs, it was found that the MXene escaped accumulation into the major organs. However, it appeared that they either excreted via urine through renal clearance pathways or retained in the tumor site through the enhanced permeability and retention (EPR) effect [7]. While incubated in hMPO and H_2_O_2_ enriched medium for 24 h, Nb_2_C NSs caused the complete enzyme-triggered degradation and disappearance of NSs [32]. It has been known that the degradation of TMDCs depends on the external environment, including oxidation, routes of administration, and pH, while studying their toxicity profile. While the transition metals evolved in TMDCs raised concerns about the risk of toxicity, the oxidants play a significant role to degrade them and subsequently eliminate rapidly from the body [86]. Mei et al. investigated translocation and correlation between the degradation and toxicity levels of polyvinylpyrrolidone modified 2H-phase MoS_2_ nanosheets (MoS_2_-PVP NSs) and found that MoS_2_ NSs demonstrated a different level of biodegradation with H_2_O_2_, catalase, and human myeloperoxidase (hMPO) in the following order: H_2_O_2_ < catalase < hMPO. The MoS_2_ NSs accumulated in the gastrointestinal organs and were excreted with the feces within 48 h of post intragastric administration. Remarkable clearance of NSs was observed due to decreased accumulation in the liver and spleen within 30 days of post intravenous injection. When the NSs were administered through the intraperitoneal route, they accumulated slightly in the spleen [122]. Chen et al. observed rapid degradation of PEGylated MoS_2_ (MoS_2_-PPEG) in neutral pH conditions and noted there was a slower degradation in the acidic tumor microenvironment. Furthermore, it was found that the major degradation product of MoS_2_-PPEG was water-soluble Mo-based ions, which were detected in the urine of mice. It was reported that MoS_2_ could be oxidized and transformed into water-soluble MoO_4_^2−^ species in the physiological environment and readily be excreted via both renal and fecal pathways [121]. TMDCs have a different level of oxidation, transformation, and excretion, depending on the elemental composition of different transition metals and their various physicochemical properties. WS_2_ NSs possess high stability and are hardly degraded in the physiological environment and hence retains in RES organs for a long time. TiS_2_ NSs are unstable, and their gradual transformation into the water-insoluble TiO_2_ aggregates slowly eliminates from the body [112,123].

As far as the toxicity of graphene-based nanomaterials is concerned, the sharp edges of graphene are the main contributor to its high cytotoxicity. The increase in active edge sites and surface area contributes to the increased cytotoxicity; however, surface modification may reduce the toxicity level [124]. The cytotoxicity of various 2D graphene is more significant than that of 2D TMDCs. Graphene family nanomaterials are generally catalyzed by oxidative enzymes such as peroxidases, and their degradation abilities are mostly dependent on the number of layers, the lateral dimension, and the C/O ratio [125]. It was reported that hypochlorite (ClO^−^), which is produced in the human body by various enzymes, such as myeloperoxidase (hMPO) and eosinophil peroxidase induce oxidative damage to the graphitic backbone. The ClO^−^ ions from NaClO completely degrade 2D GO NSs with rapid kinetics compared to 1D oxidized carbon nanotubes and nanohorns [126]. In another research work, hMPO has been shown to completely metabolize highly dispersed GO NSs compared to its action against aggregated GO [127]. Nanomaterial biodegradation and biological corona formation, collectively known as biotransformation, occurs when GO NSs are degraded for 14 days in human blood plasma [128]. Generally, MnO_2_ NSs can transform into degraded products within a few hours in the physiological environment [129]. Though MnO_2_ NSs are relatively stable under neutral and alkaline pH conditions, they easily decompose into Mn^2+^ and O_2_ at acidic pH [111]. The free Mn^2+^ ions exhibit an outstanding MRI contrast effect due to its five unpaired 3d electrons and hence poses a high relaxivity than that of MnO_2_ nanoparticles. The degradation process of MnO_2_ NSs can be monitored by MRI, due to the much-improved MRI contrast enhancement after decomposition [112]. The literature report reveals that the toxicity of Mn^2+^ is much higher than that of MnO_2_ NSs [103]. Although many studies have demonstrated that the degradation products (Mn^2+^) of MnO_2_ NSs can be excreted by renal clearance, the elimination amount is not yet precise. 

BP NSs strongly react with oxygen and water and degrade to non-harmful phosphate, and phosphonate ions since the elemental P atoms are connected by weak forces in the structure. Hence, their accumulation in the body organs is better than other 2D nanomaterials [130]. When The biodegradation rate of exfoliated BP NSs was investigated under distinct atmospheres, viz. Ar, air, and O_2_, it was discovered that the NSs degrades faster in the O_2_ atmosphere [29]. There are several reports to prove that the toxicity of Pd NSs is majorly dependent on its size. Chen et al. synthesized 2D Pd NSs with diameters ranging from 5 to 80 nm and investigated their size-dependent biodistribution, elimination, toxicity, and genomic gene expression profiles. The results showed that 5-nm Pd NSs evaded from the reticuloendothelial system with a longer blood half-life and were cleared by renal excretion, while larger sized Pd NSs mainly accumulated in the liver and spleen. Although Pd NSs did not induce any significant toxicity at the cellular level, lipid accumulation in the liver and inflammation was observed slightly in the spleen. Genomic gene expression analysis revealed that 80-nm Pd NSs interacted with more cellular components and influenced more biological processes in the liver when compared to 5-nm Pd nanosheets [59]. In another study, Li et al. reported that 5 nm Pd NSs effectively accumulated on the tumor sites of the animal models through the enhanced permeability and retention (EPR) effect and could be excreted out of rats through urine [119].

The investigations on the biosafety of 2D theranostic nanomaterials are still at an infancy stage. It is envisaged that a systematic study on the biological effects of the nanomaterials, toxicity profiles, and the relevant clinical trials would accelerate the commercialization of 2D theranostic nanomaterials soon.

## 4. Conclusions and Perspectives

The potential theranostic application of 2D nanomaterials in cancer treatment via both in vitro and in vivo has been discussed in this review. Though theranostic 2D nanomaterials were found effective, there are some issues to be resolved: (1) Many research groups have explored nanosheets as the only 2D nanostructured material without giving much importance to other nanostructured materials such as nanorings, nanoribbons, nanoplates, nanoleaves, etc. (2) Though biodistribution of nanomaterials into different organs of animals was studied, the researchers have to conduct more trials to know the target specificity of studied materials. (3) Systematic studies are required to compare the effectiveness of a specific 2D nanomaterial in various types of tumors subjecting to different animal species. The following suggestions can be considered for the future direction in this field. (1) Analyzing both in vitro and in vivo studies of different 2D nanostructured biocompatible materials. (2) Employing sophisticated techniques and methodologies, including a computational study to investigate the organ-specificity of reported theranostic materials. (3) Carrying out extensive biological studies to compare the effectiveness of nanomaterials among different cancer models. We expect that the researchers with interdisciplinary backgrounds will advance the field of cancer treatment using 2D nanomaterials and nanocomposites by reflecting the problems and the concerning suggestions.

## Figures and Tables

**Table 1:** Published in vitro and in vivo cancer treatment studies involving 2D theranostic nanomaterials.

2D Nanomaterials, Dimensions, and Their Nanocomposites	Theranostic Effects	In Vitro Cell Line/ In Vivo Animal Model, the Concentration of Nanocomposite and Biological Parameters	Biological Results	Reference
Ti_3_C_2_ NSs (PS = 227 nm, LT = 2.8 nm); MnO_x_ (LS = 15.5 nm, LT = 1.5 nm); MnO_x_/ Ti_3_C_2_-SP	pH-responsive T_1_-weighted MR imaging, PA imaging, and NIR- triggered PTT	4T1 cells; 10–160 μg/mL; Irradiation by NIR laser (808 nm, 5 min, 1.5 W/cm^2^)	(a) T_1_-weighted MR imaging revealed a concentration-dependent brightening effect and enhanced positive MRI signals in acidic conditions. (b) Efficient endocytosis into cancer cells occurred due to the smaller planar size of the composite. (c) High photothermal conversion ability elevated the intracellular temperature to ablate the cancer cells.	Dai et al., 2017 [81]
		Male BALB/c nude mice bearing 4T1 tumor; 4 groups, intravenous injection, 20 mg/kg of BW; Irradiation (808 nm, 10 min, 1.5 W/cm^2^)	A high tumor-suppressing effect was observed due to the increase in temperature from 25 °C to 60 °C under the laser irradiation.	
Ti_3_C_2_ (PS = 100 nm, ZP = −23.18 mV); hyaluronic acid capped Ti_3_C_2_-DOX	PTT, PDT, and drug delivery	HCT-116 cells; 0, 6.25, 12.5, 25, 50 and 100 μg/mL of Ti_3_C_2_ and 0, 5.25, 10.5, 21, 42, and 84 μg/mL of DOX; Irradiation (808 nm, 10 min, 0.8 W/cm^2^)	Low dark toxicity and a dose-dependent photothermal killing efficiency have been observed.	Liu et al., 2017 [95]
		Athymic nude mice bearing HCT-116 tumor; 5 groups, intravenous injection, Ti_3_C_2_-DOX (DOX dose: 1.6 mg/kg of BW and Ti_3_C_2_ dose: 2.0 mg/kg of BW); Irradiation (808 nm, 10 min, 0.8 W/cm^2^)	(a) An intense fluorescent signal of DOX was detected in the tumor site of Ti_3_C_2_-DOX injected mice, while a much lesser signal was detected in the liver and kidney. (b) Due to the accumulation of Ti_3_C_2_-DOX into the tumor, the rise in temperature was from 34.0 °C to 53.1 °C.	
Ti_3_C_2_ (PS = 120 nm, LT = 0.9 nm); DOX@Ti_3_C_2_-SP (ZP = −28.9 mV, D_H_ = 164.2 nm)	PTT and pH-responsive drug delivery	4T1 cells; 0, 38, 75, 150, 300 and 600 μg/mL; Irradiation (808 nm, 1.5 W/cm^2^, 5 min)	(a) DOX@Ti_3_C_2_-SP+laser showed a higher killing effect compared to Ti_3_C_2_-SP+laser, DOX@Ti_3_C_2_-SP, DOX only, laser only, and control. (b) At 300 μg/mL of the nanocomposite, the temperature in the tumor microenvironment increased up to 52 °C. (c) Upon irradiation, DOX releasing percentage from the nanocarrier at pH 4.5 (81.5%) was larger than that from the carrier at pH 6.0 (51.5%) and 7.4 (33.2%).	Han et al., 2018 [28]
		4T1 breast tumor-bearing female BALB/c nude mice; 5 groups, 15 mg/kg (BW), intravenous injection; Irradiation (808 nm, 1.5 W/cm^2^, 10 min)	(a) The modified Ti_3_C_2_ effectively resided into tumor tissue through the EPR effect with the accumulation ratio of 2.0 % at the initial stage (4 h) and 3.6% with the prolonged duration (24 h). (b) The temperature was induced by irradiation to 68.5 °C, which is enough to ablate the tumor. (c) DOX@Ti_3_C_2_-SP+laser achieved a complete tumor eradication without re-occurrence, while other groups had a remaining volume of cancer.	
Ti_3_C_2_ (PS = 200 nm, LT = 2 nm); Ti_3_C_2_@Au-PEG	PTT/RT and PA/CT dual-modal imaging	4T1 cells; 50 μg/mL; Irradiation (PTT—1064 nm, 0.4, 0.6 and 0.75 W/cm^2^, 5 min; CT-X-ray- 6 Gy)	(a) 4T1 cells treated with Ti_3_C_2_@Au-PEG+laser showed an effective ablation of tumor cells than Ti_3_C_2_-PVP nanosheets, comparatively. (b) Upon irradiation, the temperature in solution was enhanced by 36.4 °C within 5 min.	Tang et al., 2019 [96]
		Female BALB/c mice bearing 4T1 tumor; 6 groups, intravenous injection, 20 mg/kg (BW); Irradiation (PTT—1064 nm, 0.75 W/cm^2^, 10 min; CT- 6 Gy)	(a) Mild PTT could overcome tumor hypoxia by increasing blood flow in the blood vessels. (b) PTT+X-ray therapy healed the tumor with significant inhibition efficiency than PTT therapy alone.	
Nb_2_C; CTAC@Nb_2_C-MSN-PEG-RGD (D_H_ = 220.2 nm)	PTT/PA and RGD targeted drug-delivery	U87 cells; 31, 61.5, 125, 250, and 500 μg/mL; Irradiation (1064 nm, 1.5 W/cm^2^, 5 min)	(a) CTAC@Nb_2_C-MSN-PEG-RGD+laser induced cancer cell apoptosis and death significantly higher than CTAC@Nb_2_C-MSN-PEG-RGD without laser. (b) The photothermal conversion efficiency of the nanocomposite was 28.6%. (c) The increase in temperature was from 30 °C to 65 °C at 500 μg/mL.	Han et al., 2018 [97]
		U87 tumor-bearing female nude mice; 4 groups, intravenous injection, 15 mg/kg (BW); Irradiation (1064 nm, 1.5 W/cm^2^, 10 min)	(a) The nanocomposite accumulated into the tumor site via RGD recognition with targeting efficacies of 5.47%, 9.57%, and 5.75% at 2 h, 4 h, and 24 h of post-intravenous injection, respectively. (b) The surface tumor temperature induced by irradiation reached 52.3 °C at 10 min. (c) CTAC@Nb_2_C-MSN-PEG-RGD+laser showed significantly higher tumor inhibition efficiency (92.37%) than CTAC@Nb_2_C-MSN-PEG-RGD without laser (35.96%).	
Nb_2_C (PS = 150 nm, LT = 0.3–0.8 nm); Nb_2_C-PVP	PTT (NIR-I and NIR-II biowindows) and PA	4T1 or U87 cancer cells; 100 μg/mL; Irradiation (NIR I—808 nm, NIR II—1064 nm, 0.5–2.0 W/cm^2^, 5 min)	(a) At 40 μg/mL of Nb_2_C exposure, the temperature of the solution increased up to 60 °C at 1.5 W/cm^2^. (b) Cell death increased depending on laser density.	Lin et al., 2017 [32]
		Female Kunming mice bearing 4T1 tumor. 4 groups, intravenous injection, 20 mg/kg; Irradiation (NIR I—808 nm, 10 min; NIR-II—1064 nm, 10 min)	The tumor-site temperature in mice treated with the nanocomposite increased from 30 °C to 61 °C for NIR I and from 30 °C to 65 °C for NIR II.	
TiS_2_ (PS = 100 nm); TiS_2_-PEG	PTT (NIR-I or NIR-II) and PA imaging	4T1 cells; 12.5, 25, 50, and 100 μg/mL; Irradiation by NIR laser (808 nm, 0.8 W/cm^2^, 5 min)	The concentration-dependent cell-killing effect was observed by the influence of TiS_2_-PEG nanosheets under irradiation.	Qian et al., 2015 [37]
		Female BALB/c mice bearing 4T1 tumor; 4 groups, intravenous injection, 20 mg/kg; Irradiation (808 nm, 0.8 W/cm^2^, 5 min)	The tumor in the mice group (TiS_2_-PEG+laser irradiation) was completely ablated, and no regrowth was observed. Tumor in control groups showed rapid growth after treatment.	
MoS_2_ (LT = 0.8–1.0 nm); MoS_2_-CS-DOX	PTT, IR thermal imaging, CT signaling, and drug delivery	KB and Panc-1 cells; 0–100 μg/mL; Irradiation (808 nm, 1.0 W/cm^2^, 8 min)	The combination of hyperthermia and chemotherapy effectively released DOX into the cells facilitating cell-killing ability.	Yin et al., 2014 [98]
		Male BALB/c nude mice bearing Panc-1 cells; 5 groups, intratumor injection, 2.0 mg/kg; Irradiation (PTT—808 nm, 0.5, 0.7 and 0.9 W/cm^2^, 7 min; CT X-ray- 0–40 mg/mL of MoS_2_-CS, 70 kV and 100 μA)	The temperature of the tumors on the MoS_2_-CS-DOX injected mice rapidly elevated by ΔT = 22.5 °C to kill the cancer tumor.	
MoSe_2_; MoSe_2_ (Gd^3+^-3), PS = 100–150 nm, LT = 1.8 nm); MoSe_2_(Gd^3+^-3)-PEG	MR/PA bimodal imaging and PTT	Hep G2 cells; 0–15 μg/mL; Irradiation (808 nm, 2.0 W/cm^2^, 5 min)	Upon irradiation, the temperature of deionized water was elevated by 2.9 °C whereas the solution of MoSe_2_(Gd^3+^-3)-PEG was increased by >30 °C	Pan et al., 2018 [78]
		Hep G2 tumor-bearing BALB/c nude mice; 4 groups, intravenous injection, 1.0 mg/kg; Irradiation (808 nm, 2 W/cm^2^, 5 min)	(a) The measurement of Mo^4+^ concentration in tissue lysate using ICP-AES revealed that PEG coating in MoSe_2_(Gd^3+^)-PEG nanocomposite treated mice led to the superior tumor accumulation and prolonged blood circulation. (b) The theranostic effects of the nanocomposite caused the ablation of tumors leaving black scar only at the tumor site at 14 days of post-treatment.	
Bi_2_Te_3_; BSA-Bi_2_Te_3_, PS = 100 nm, LT = 15 nm; BSA-Bi_2_Te_3_/MB NSs.	PDT/PTT	HeLa; BSA-Bi_2_Te_3_/MB NSs (200 μg/mL of BSA-Bi_2_Te_3_ and 10 μg/mL of MB); Irradiation (PDT—650 nm, 50 mW/cm^2^, 15 min; PTT—808 nm, 2.0 W/cm^2^, 5 min)	Intracellular ROS was produced largely inside the cancer cells under irradiation by the combination therapy.	Bai et al., 2018 [38]
		U14 tumor-bearing Kunming female mice; 5 groups, intravenous injection, 200 μL of BSA-Bi_2_Te_3_ (200 μg/mL) and MB (10 μg/mL); Irradiation (PDT—650 nm, 50 mW/cm^2^, 15 min; PTT—808 nm, 2.0 W/cm^2^, 10 min)	The tumors in mice of BSA- Bi_2_Te_3_/MB group were eliminated without recurrence after 15 days of treatment.	
NGO (PS = 100–500 nm, LT = 1 nm); PEG-BPEI- functionalized, IR-808-conjugated NGO sheets (NGO-808)	PDT/PTT	A549 and Lewis lung cancer cells; NGO-PEG-BPEI (0–30 μg/mL), IR-808 (0–10 μM) and NGO-808 (0–10 μM); Irradiation (808 nm, 2 W/cm^2^, 5 min)	(a) The dual functionalization (PEG-BPEI) had promoted a higher cellular uptake of NGO-808. (b) Generation of singlet oxygen is higher in NGO-808 treated cells than that in NGO-PEG-BPEI and blank PBS.	Luo et al., 2016 [43]
		A549 or Lewis tumor-bearing C57BL/6 athymic male nude mice; 6 groups, intravenous injection, 10 mg/kg of NGO-808, 2 mg/kg of IR-808, and 8 mg/kg of NGO-PEG-BPEI; Irradiation (808 nm, 1 W/cm^2^, 5 min)	(a) NIR fluorescence imaging enabled tumors to be visualized at 48 h of post-intravenous injection. (b) The surface temperature of cancer in NGO-808+NIR treated mice was increased to 59–62 °C, which was enough to ablate malignant cells.	
NGO (size = 38.4 ± 3.1 nm, ZP = −54.9 ± 7.1 nm); Pluronic F127- NGO-MB	PDT/PTT, IR thermal imaging and pH-dependent drug release	NIH-3T3 and HeLa cells; Pluronic F127- NGO-MB (10 μg/mL of NGO and 2 μg/mL of MB); Irradiation (PDT—655 nm, 150 mW/cm^2^, 3 min; PTT—808 nm, 2 W/cm^2^, 3 min)	(a) The temperature level of solution arose rapidly from 28.5 °C to 45.5 °C at 3 min of irradiation. (b) Higher cellular uptake and rapid release of MB from the nanocomposite inside the cells resulted in an enhanced therapeutic effect.	Sahu et al., 2013 [99]
		HeLa tumor-bearing athymic male nude mice; 4 groups, intravenous injection, 10 mg/kg of NGO and 2 mg/kg of MB; Irradiation (PDT—650 nm, 150 mW/cm^2^, 10 min; PTT—808 nm, 2 W/cm^2^, 3 min)	In Pluronic F127- NGO-MB mice group, dual therapy (PDT-PTT) caused a complete ablation of tumor tissues at 15 days of post-treatment.	
NGO (PS of PEG-NGO = 900 nm, LT = 4 nm); PEG-NGO-C225/EPI	PTT and tumor-targeted chemotherapy	U87 cells; 0–25 μg/mL of EPI in nanocomposite; Irradiation (808 nm, 2 W/cm^2^, 2 min)	(a) The nanocomposite elevated the temperature of the solution from 36 °C to 94 °C. (b) Breakage in double-strands of DNA occurred in tumor cells at about 70 °C.	Yang et al., 2013 [100]
		U87 tumor-bearing mice; 5 groups, intravenous injection, 6 mg/kg; Irradiation (808 nm, 2 W/cm^2^, 2 min)	A total tumor ablation was achieved in PEG-NGO-C225/EPI+NIR treated mice group in 10 days of treatment.	
NGO (size = 200–600 nm); GO-IONP-Au-PEG	MR, X-ray, IR thermal imaging and magnetically assisted PTT	4T1 cells; 10 μg/mL of GO in nanocomposite; Magnetic field under the center of cell culture dish for 2 h followed by NIR irradiation (808 nm, 1 or 2 W/cm^2^, 5 min)	Fluorescence images revealed that the cells near the magnet were effectively killed, while those far from the magnetic field were not affected.	Shi et al., 2013 [101]
		Female 4T1 tumor-bearing BALB/c mice; 3 groups, intratumoral injection, 50 μg/mL of GO in nanocomposite; Irradiation (808 nm, 0.75 W/cm^2^, 5 min)	(a) During irradiation, the GO-IONP-Au-PEG treated mice group witnessed an increase in temperature up to 55 °C, whereas GO-PEG or PBS showed up to 45 °C and 38 °C, respectively. (b) Effective ablation of the tumor was observed at 6 days of post-treatment.	
NGO (PS of NGO-PEG = 14 nm, LT = 1.30 ± 0.55 nm); NGO-PEG-DVDMS	PDT/PTT, fluorescence, PA, and IR thermal imaging	PC9 cells; NGO-PEG-DVDMS aqueous solution (1 μg/mL of NGO-PEG and 2 μg/mL of DVDMS); Irradiation (PDT—630 nm, 5 J; PTT—808 nm, 1 W/cm^2^, 5 min)	NGO-PEG-DVDMS showed a high photothermal conversion effect with a temperature point reaching up to 60.2 °C, which was significantly higher than that of NGO-PEG (49.1 °C).	Yan et al., 2015 [79]
		PC9 tumor-bearing athymic nude mice; 4 groups, intravenous injection, 200 μL of GO-PEG-DVDMS (1.0 mg/kg of GO-PEG); Irradiation (PDT—630 nm, 50 J; PTT—808 nm, 1 W/cm^2^, 10 min)	The tumor temperature in GO-PEG-DVDMS treated mice increased up to 57 °C to eradicate the tumor cells.	
NGO (PS of NGO- PEG = <50 nm, LT = 1.5 nm); NGO-PEG-HPPH	PDT, fluorescence, and PET imaging	4T1 cells; 0.49 μg/mL; Irradiation (PDT—671 nm, 2–8 W/cm^2^, 3 min)	Cells treated with GO-PEG-HHPH exhibited a stronger fluorescence intensity and presented a higher cell death than those treated with free HPPH.	Rong et al., 2014 [102]
		4T1 tumor-bearing athymic nude mice; 6 groups, intravenous injection, 200 μL of GO-PEG-HPPH (1.0 mg/kg of HPPH and 0.77 mg/kg of GO-PET); Irradiation (671 nm, 75 mW/cm^2^, 20 min)	High tumor selectivity was observed in GO-PEG-HPPH treated mice, which was inferred from vigorous fluorescence intensity within tumor tissue rather than the liver and spleen.	
NGO (PS of PEG-NGO = 100 nm); NGO-UCNP-Ce6	PDT/PTT, UCL, and IR thermal imaging	HeLa cells; 25–800 μg/mL; Irradiation (808 nm, 0.72 W/cm^2^)	(a) When exposed to a laser, the NUC presented significant dark toxicity to HeLa cells at a concentration of 800 μg/mL.	Gulzar et al., 2018 [42]
		U14 tumor-bearing mice; 4 groups, intravenous injection; Irradiation (808 nm, 10 min)	(a) No abnormal decrease in body weight with a prolonged time. (b) Due to the combination of PTT and PDT treatment, the tumor in NGO-UCNP-Ce6 treated mice displayed an exceptional reduction in size.	
MnO_2_ nanosheets (D_H_ = 80 nm); Ce6@MnO_2_-PEG	PDT and T_1_-MRI	4T1 cells; Ce6@ MnO_2_-PEG (0–6 μM of Ce6 and 0, 7.5, 15, 30, 60, and 90 μM of MnO_2_); Irradiation (660 nm, 5 mW/cm^2^, 30 min)	Cell killing efficiency of nanocomposite was found relatively higher in the N_2_ atmosphere than that in the O_2_ atmosphere.	Zhu et al., 2016 [103]
		4T1 tumor-bearing BALB/c female nude mice; 4 groups, intravenous injection, Ce6@ MnO_2_-PEG (1 mg/mL of MnO_2_ and 0.45 mg/mL of Ce6); Irradiation (661 nm, 5 mW/cm^2^, 1 h)	The mice treated with the nanocomposite showed a significantly decreased tumor hypoxia, which led to effective PDT therapy.	
MnO_2_ nanosheets (LT = 2 nm, PS = 255 nm); MnO_2_-SPs	PTT, T_1_-MRI, and pH sensitive drug release	4T1 cells; (0, 37.5, 75, 150, 300, and 600 μg/mL); Irradiation (808 nm, 1.5 W/cm^2^, 5 min)	Confocal laser scanning microscope image observations showed a strong red fluorescence indicating a significant cell death in MnO_2_-SPs+laser group.	Liu et al., 2018 [51]
		4T1 tumor-bearing BALB/c female nude mice; 4 groups, intravenous injection, 100 μL of MnO_2_-SPs (600 μg/mL); Irradiation (808 nm, 1.5 W/cm^2^, 5 min)	The tumor surface temperature of the MnO_2_-SPs+laser group elevated from 37 °C to 57 °C, while the heat of the laser-only group just increased by 1 °C.	
MnO_2_ nanosheets; MnO_2_ NSs anchored with upconversion nanoprobes (UCSMs)	PDT, RT, UCL and PA imaging	Hc-4T1 cells; 0–200 μg/mL; Irradiation (1.5 W/cm^2^, 5 min); X-ray (5 Gy, 5 min)	UCL intensity significantly enhanced in the cytoplasm of hc-4T1 cells owing to the decomposition of MnO_2_ nanosheets.	Fan et al., 2015 [50]
		Hc-4T1 tumor-bearing BALB/c female nude mice; 7 groups, intratumor injection, UCSMs (8 mg/mL); Irradiation (2 W/cm^2^, 10 min); X-ray (8 Gy, 5 min)	UCSMs+RT+NIR promoted a synergetic PDT/RT effect mostly on 4T1 solid tumors causing a remarkable anti-tumor efficacy.	
BP nanosheets (LT of p-BPNSs = 1.3 nm, D_H_ = <200 nm, ZP = −26.1 mV); RP-p-BPNSs	PTT, PA imaging and targeted delivery	LO2, MCF-7, and A549 cells; 0–200 μg/mL; Irradiation (808 nm, 1.0 W/cm^2^, 10 min)	The cell viability of A549 cells (52%) treated with the nanocomposite significantly reduced when compared to that of MCF-7 cells (62%).	Li et al., 2019 [104]
		A549 tumor-bearing BALB/c nude mice; 5 groups, 1 mg/mL of RP-p-BPNSs, intravenous injection; Irradiation (808 nm, 1.0 W/cm^2^, 10 min)	(a) The efficient tumor accumulation of RGD peptide-modified RP-p-BPNSs increased PA intensity dramatically. (b) The tumor site temperature rapidly increased by 23.9 °C within 10 min, and finally reached the temperature of about 56.4 °C to induce local hyperthermia.	
BP nanosheets (LS = 200–300 nm, LT = 5.3 nm,); BP-R-D@PDA-PEG-Apt	PTT, IR thermal imaging, gene delivery and targeted drug delivery	MCF-7 and MCF-7/ADR cells; BP-R-D@PDA-PEG-Apt, 0.5–10 μg/mL of BP; Irradiation (808 nm, 1.0 W/cm^2^, 10 min)	DOX-siRNA-BP caused a higher cytotoxicity in MCF-7/ADR cells. For DOX alone, the inhibition ratio of cells was about <20% up to 10 μg/mL.	Zeng et al., 2018 [54]
		MCF-7/ADR tumor-bearing SCID female mice; 7 groups, BP-R-D@PDA-PEG-Apt (5 mg/kg of DOX), intravenous injection; Irradiation (808 nm, 1.5 W/cm^2^, 5 min)	BP-R-D@PDA-PEG-Apt+NIR treated group led to tumor necrosis, causing severe destruction to tumor cells.	
BP nanosheets (LT = 1 nm, D_H_ = 220 nm); BP-PEI-siRNA	PTT and gene therapy	MCF-7 cells; BP-PEI-siRNA (25 μg/mL of BP-PEI and 200 nM of siRNA); Irradiation (808 nm, 1.0 W/cm^2^, 10 min)	(a) A strong red fluorescence observed in CLSM images indicated that the cells treated with BP-PEI-siRNA promoted siRNA internalization. (b) The cell growth inhibition rate reached 64% under irradiation.	Wang et al., 2018 [56]
		MCF-7 tumor-bearing female BALB/c mice; 4 groups, BP-PEI-siRNA (10 mg/kg of BP-PEI and 1 mg/kg of siRNA), intratumor injection; Irradiation (808 nm, 1.0 W/cm^2^, 10 min)	The theranostic effects of the nanocomposite not only ablated the tumor but also suppressed tumor growth during the observation period.	
BP nanosheet (PS = 120 nm, LT = 24.3 nm); BP@PDA-Ce6&TPP	PDT, fluorescence imaging, and organelle-targeting drug-delivery	HeLa cells; BP@PDA-Ce6&TPP solution (1–25 μg/mL of BP@PDA); Irradiation (660 nm, 0.5 W/cm^2^, 5 min)	(a) 50% of the treated cells were killed in the BP@PDA-Ce6&TPP+laser group, which were larger than those treated with BP@PDA-Ce6 (32%) and BP@PDA (3%). (b) Hela cell uptake was higher in mitochondria than in the cytosol.	Yang et al., 2019 [55]
		HeLa tumor-bearing female nude mice; 5 groups, 0.56 mg/kg of BP@PDA in nanocomposite, intravenous injection; Irradiation (660 nm, 0.5 W/cm^2^, 10 min)	A more substantial tumor eradication efficiency was observed in the tumor of the BP@PDA-Ce6&TPP+laser treated mice group.	
BP nanosheets (PS 400 nm, D_H_ = 344.6 nm); BP-DLH (doxorubicin (D), poly-L-lysine (L), and hyaluronic acid (H))	PTT, pH- and targeted drug delivery	MCF-7 and MDA-MB-231; 0.001–50 μg/mL; Irradiation (808 nm, 0.8 W/cm^2^, 2.5 min)	At 50 μg/mL, >95% of cell cytotoxicity was observed in both the cell lines upon irradiation.	Poudel et al., 2018 [105]
		MDA-MB-231 xenograft-bearing BALB/c mice; 5 groups, 5 mg/kg. intravenous injection; Irradiation (808 nm, 3 W/cm^2^, 3 min)	(a) Tumor temperature increased up to 48.8 °C after 5 min. (b) At the end of the experiment (24 days), the mean tumor volume of the treated mice was in the following order as BP-DLH+NIR<BP-LH<free doxorubicin<BP-LH+NIR<BP-LH<control (untreated).	
Pd nanosheets (size = 4.5 nm); Pd-PEI-Ce6	PTT/PDT	HeLa cells; Pd-PEI-Ce6 (50 μg/mL of Pd and 2.77 μg/mL of Ce6); Irradiation (660 nm, 0.5 W/cm^2^, 5 min)	Only 50% of cells survived in Pd-PEI-Ce6 treated cells.	Zhao et al., 2014 [106]
		S180 bearing female Kunming mice; 4 groups, intratumor injection, Pd-PEI-Ce6 (50 μg/mL of Pd and 2.77 μg/mL of Ce6); Irradiation (660 nm, 0.5 W/cm^2^, 5 min)	The tumor temperature increased quickly from 35 °C to 52 °C in Pd-PEI-Ce6 treated mice group after 5 min of laser exposure, and the tumor was eradicated after 7 days of treatment.	
Ultrasmall Pd nanosheets (SPNS) (size = 4.4 nm, ZP = −17.7 mV); SPNS-DOX-GSH	PTT and chemotherapy	QGY-7703 cells; 20 μg/mL of SPNS-DOX solution (0, 7.5, 15 and 30 ppm Pd); Irradiation (808 nm, 1.4 W/cm^2^, 2 min)	The temperature of the SPNS-DOX solution containing 30 ppm Pd nanosheets elevated from 25.6 °C to 48.8 °C at 10 min of irradiation.	Tang et al., 2015 [107]
		4T1 tumor-bearing female BALB/c mice; 7 groups, intravenous injection, SPNS-DOX-GSH (1.5 mg/mL); Irradiation (808 nm, 0.3 W/cm^2^, 5 min)	Tumor temperature increased from 32 °C to 58.5 °C within 5 min of irradiation.	
Pd (size of Pd@Ag nanoplates = 41 nm); Pd@Ag@*m*SiO_2_-Ce6	PTT/PDT	HeLa cells; 90 or 120 μg/mL; Irradiation (660 nm, 0.1 W/cm^2^, 5 min or 808 nm, 1 W/cm^2^, 10 min)	(a) Irradiation with 808 nm followed by 660 nm laser or simultaneous irradiation established an increased cell death at all the concentrations investigated. (b) The rise in temperature was from 27 °C to 38.8 °C	Shi et al., 2013 [108]
		S180 tumor-bearing female Kunming mice; 5 groups, intratumor injection, 150 μg/mL; Irradiation (660 nm, 0.1 W/cm^2^, 5 min or 808 nm, 1 W/cm^2^, 5 min)	The tumor temperature increased from 27 °C to 43 °C.	
Au nanoring (thickness = 2–20 nm, size = 25, 50, and 130 nm); HS-PEG@Au	PTT, PET and PA imaging	Raw 264.7 cells; 0.037 nM of 50 nm Au nanoring; Irradiation (808 nm, 0.5 W/cm^2^)	Thicker Au nanorings showed better photothermal stability than the thinner ones.	Liu et al., 2017 [109]
		U87MG tumor-bearing female nude mice; 4 groups, intravenous injection, 100 μL of ^64^Cu labeled 50 nm Au nanoring; Irradiation (808 nm, 0.75 W/cm^2^, 5 min)	Due to the accumulation of Au nanorings in tumors, strong fluorescent signals were observed.	
B nanosheets (LS = 3 nm, PS = 110 nm); B-PEG/DOX NSs	PTT, PA, IR thermal, fluorescence imaging, and drug release	MCF-7 and PC3 cells; B-PEG/DOX NSs (0–88 μg/mL of B and 0–100 μg/mL of DOX); Irradiation (808 nm, 1.0 W/cm^2^, 5 min)	(a) Under irradiation, B-PEG NSs treated cells showed dose-dependent toxicity. (b) Over 95% of cell death was observed at a DOX concentration of 100 μg/mL.	Ji et al., 2018 [110]
		MCF-7 tumor-bearing female nude BALB/c mice; 5 groups, intravenous injection, B-PEG/DOX NSs (5.3 mg/kg of B, 6 mg/kg of DOX); Irradiation (808 nm, 1.0 W/cm^2^, 10 min)	After 14 days of treatment, the tumors disappeared without recurrence in B-PEG/DOX NSs+NIR treated mice.	

Abbreviations: NSs nanosheets, Ti_3_C_2_ titanium carbide, TiS_2_ titanium disulfide, MnO_2_ manganese dioxide, MoS_2_ molybdenum disulfide, Nb_2_C niobium carbide, MoSe_2_ molybdenum diselenide, Bi_2_Te_3_ bismuth telluride, EPI epirubicin, DOX doxorubicin, Gd gadolinium, PS planar size, LT layer thickness, ZP zeta potential, D_H_ hydrodynamic diameter, SP soybean phospholipid, CTAB cetanecyltrimethylammonium chloride, MSN mesoporous silica-coated nanocomposite, PEG polyethylene glycol, c(RGDyC) cyclic arginine-glycine-aspartic pentapeptide, PVP polyvinylpyrrolidone, PTT photothermal therapy, PDT photodynamic therapy, PA photoacoustic, MR magnetic resonance, CS chitosan, MB methylene blue, BSA bovine serum albumin, BPEI polyethylenimine, NGO nanographene oxide, EGFR epidermal growth factor receptor, PET positron emission tomography, UCL upconversion luminescence, UCNP upconversion nanoparticles, Ce6 chlorin e6, T_1_-MRI T_1_-weighted MR imaging, RT radiation therapy, BP black phosphorous, HCT-116 human colon cancer cells, KB human epithelial carcinoma, Panc-1 human pancreatic cells, Hep G2 human liver carcinoma cells, NIH-3T3 mouse embryonic fibroblast cells, hc-4T1 hypoxic murine breast cancer cells, U14 murine hepatocarcinoma, LO2 human hepatocyte cell line, MCF-7 human breast cancer cells, MCF-7/ADR multidrug-resistant breast cancer cell, A549 adenocarcinomic human alveolar basal epithelial cell line, MDA-MB-231 human breast adenocarcinoma, QGY-7703 human hepatoma cells, Raw 264.7 mouse leukemic monocyte macrophages, QSG-7701 human paratumor cirrhosis hepatocellular cell line, PC3 human prostatic cancer cells, PC9 human lung adenocarcinoma, U87MG human glioblastoma astrocytoma cells, S180 murine sarcoma cells, HeLa human cervical cancer cells, PEI polyethyleneimine, SH thiol, IONP iron oxide nanoparticles, HPPH 2-(1-hexyloxyethyl)-2-devinyl pyropheophorbide-alpha, p-BPNSs 1-pyrenebutyric acid modified BP nanosheets, PDA polydopamine, Apt aptamers, TPP triphenyl phosphonium, GSH glutathione, NIR near Infra-red radiation, CT computed Tomography, BW body weight, SCID severe combined immunodeficient, DVDMS sinoporphyrin sodium.
